# Combined effects of canola plant density and insecticide management strategies on flea beetle abundance, canola defoliation, and yield across the Canadian prairies

**DOI:** 10.1002/ps.70320

**Published:** 2025-10-30

**Authors:** Shayla Woodland, Héctor A Cárcamo, Tyler Wist, Jennifer Otani, John Gavloski, Rob W Duncan, Tharshinidevy Nagalingam, Alejandro C Costamagna

**Affiliations:** ^1^ Department of Entomology University of Manitoba Winnipeg MB Canada; ^2^ Agriculture and Agri‐Food Canada Lethbridge Research and Development Centre Lethbridge AB Canada; ^3^ Agriculture and Agri‐Food Canada Saskatoon Research and Development Centre Saskatoon SK Canada; ^4^ Agriculture and Agri‐Food Canada Beaverlodge Research Farm Beaverlodge AB Canada; ^5^ Manitoba Agriculture Carman MB Canada; ^6^ Department of Plant Science University of Manitoba Winnipeg MB Canada

**Keywords:** canola, flea beetles, integrated pest management, neonicotinoids, plant density, yield

## Abstract

The crucifer flea beetle, *Phyllotreta cruciferae* (Goeze), and the striped flea beetle, *Phyllotreta striolata* (Fabricius) (Coleoptera: Chrysomelidae), are devastating pests of canola (*Brassica napus* (L.)) in North America. Currently, farmers rely on prophylactic, neonicotinoid insecticide‐coated seed to control them. This results in most fields being treated with insecticides regardless of flea beetle levels. Moreover, if emergence of canola or seedling growth is delayed, or under very high flea beetle populations, seed treatments alone can fail to suppress damage and require foliar insecticides or reseeding. We conducted 15 replicated field trials in four regions of the Canadian prairies testing the effects of three canola planting densities combined with two flea beetle management treatments (seed treatment and foliar spray) and two controls (‘flea beetle‐free’ treatment and untreated control) from 2018 to 2021. Although flea beetles increased as plant density increased, as predicted by the resource concentration hypothesis, this was offset by a dilution at the plant level and critically, defoliation levels did not increase at high plant densities. Using seed treatments as a management strategy generally produced similar results to using only foliar sprays at the 25% injury threshold when considering flea beetles per plant and yield. More importantly, yield increased with increased plant density regardless of flea beetle abundance, management treatment, and region. We conclude that increasing plant density is a sustainable technique that may be combined with other strategies to protect canola yield, but finding an economical plant density to protect yield from flea beetle damage will require further research. © 2025 His Majesty the King in Right of Canada and The Author(s). *Pest Management Science* published by John Wiley & Sons Ltd on behalf of Society of Chemical Industry. Reproduced with the permission of the Minister of Agriculture and Agri‐Food.

## Graphical abstract

Increasing canola plant density reduced flea beetle abundance per plant and increased yield, independent of region or insecticide use, highlighting its potential as a component of integrated pest management.

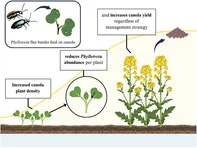

## 1 INTRODUCTION

Flea beetles (Coleoptera: Chrysomelidae) are major pests of several *Brassica* crops around the world.^1^ In particular, flea beetles of the genus *Phyllotreta* are some of the most important pests of canola across northern Europe^2^, ^3^ and North America, and if not controlled, they are responsible for more than 10% of canola yield loss each year in North America.^4^, ^5^ The two common species found across the Canadian prairies are the crucifer flea beetle, *Phyllotreta cruciferae* (Goeze), and the striped flea beetle, *P. striolata* (Fabricius). Peak emergence of overwintered adult flea beetles happens early in the growing season, which coincides with the vulnerable seedling stage of canola, potentially causing defoliation to the growing crop and reducing yield significantly.^4^ Feeding damage from *Phyllotreta* species has been estimated to cause approximately $160.3 million Canadian dollars^6^ to $300 million USD in losses in North America each year.^7^ Due to the high economic losses these pests can cause, management practices are vital to protect the growing canola plants.

The current management practices for flea beetles in North America focus mainly on the use of systemic insecticide‐coated seed (typically with neonicotinoids) in 99.5% of canola crops in the Canadian prairies.^8^, ^9^ Repeated neonicotinoid use can lead to unintended negative effects on natural enemies and pollinators^5^, ^9^, ^10^, ^11^ and other organisms in the environment.^12^ The use of neonicotinoid insecticides as seed treatments has been banned in the European Union^13^ and is restricted or limited in some provinces in eastern Canada, specifically Ontario and Quebec in fruit crops, corn, and soybean, where there are substantive requirements for agronomic justification in order to protect pollinators.^14^, ^15^ Western Canadian provinces have re‐evaluated the use of key neonicotinoids (i.e., imidacloprid, clothianidin, and thiamethoxam) and concluded that many seed treatments remain acceptable under the condition of risk mitigation measures, although regulatory reviews are ongoing.^15^ Nevertheless, there is still pressure to reduce their use and seek environmentally friendly pest control methods.^16^

In addition to insecticide‐coated seed, flea beetle management also relies on foliar insecticides applied to canola to target feeding adults. Foliar insecticides should be applied when the defoliation reaches 25% plant area as an average injury level across the field to maintain the yield potential while minimizing the number of pesticide applications.^3^, ^17^ In Sweden, Lundin^2^ found that an injury level of 25% may be too high, especially with the inexpensive control method of foliar pyrethroid spraying. Lundin's economic injury level, using various crop prices and insecticidal treatments, ranged from 8% to 16%, which may result in more foliar applications per season. In contrast, the higher 25% injury threshold currently used in Canada may delay unnecessary sprays, but foliar applications could still result in unwanted detrimental effects, including harm to beneficial insects^18^ and the development of pest resistance,^19^ as all currently registered foliar insecticides for flea beetles in canola in Canada are broad‐spectrum rather than flea beetle‐specific.

Integrated pest management (IPM) strategies are needed to reduce the dependence of commercial canola production on insecticide management. Effective and sustainable control of pest populations in an agriculture system may stem from the management of landscape composition, including manipulating edge density or availability of host plants.^20^ These factors can influence pest populations through bottom‐up processes by altering resource availability, and through top‐down processes by affecting natural enemy communities. At a crop scale, bottom‐up manipulations can promote a system to disrupt pest life cycles and reduce pest survival, change pest behaviour, and affect pest interactions with their host plants.^21^ Bottom‐up effects are considered important factors to enhance IPM and often include common cultural control methods such as tillage, cover crops and floral strips. Other cultural control methods such as increasing the seeding rate have been successful in previous studies to reduce flea beetle defoliation in canola but are more successful in conjunction with insecticide control.^22^, ^23^, ^24^ A dilution effect, where there will be less damage to each plant due to a greater amount of canola tissue to flea beetle ratio, has been hypothesized to explain the reduction in canola damage observed at increasing seeding rate.^5^, ^23^ By contrast, the resource concentration hypothesis predicts that there would be a greater abundance of flea beetles in more dense patches of host plants,^25^ which could result in similar or higher number of flea beetles per plant than in low density plant patches. However, studies manipulating plant density and simultaneously quantifying flea beetle abundance, canola damage, and yield have not been conducted to determine the relative importance of these mechanisms and their effect on flea beetle IPM.

This study investigated the bottom‐up effects of manipulating seeding rate under different management practices on flea beetles and their damage to canola over multiple years and regions across the Canadian prairies. Specifically, we report on flea beetle abundance (2208 sticky traps), canola defoliation (137 411 assessments), and yield (695 plots) to test how flea beetle management practices such as foliar spray at the 25% defoliation level and seed‐coated neonicotinoid treatments in conjunction with three seeding rates affect (1) flea beetle abundance at the patch level, (2) flea beetle pressure at the plant level, (3) defoliation levels, and (4) canola yield. We predicted that (1) flea beetle abundances will be greater while (2) flea beetle pressure per plant will be lower in plots with a higher plant density compared to a lower density. We also predicted that lower flea beetle pressure per plant (3) will lower defoliation levels and (4) increase yield in plots with a higher plant density.

## 2 MATERIALS AND METHODS

### 2.1 Study sites and agronomic practices

This study was conducted across three major Canadian ecoregions that encompasses the majority of canola production: (1) the Lake Manitoba Plain in the prairies ecozone (Carman, MB; 49° N, 98° W); (2) Moist Mixed Grasslands in the prairies ecozone, which includes a southwest location (Lethbridge, AB; 49° N, 112° W) and a northern location (Saskatoon, SK; 52° N, 106° W); and (3) the Peace Lowland in the Boreal Plains ecozone (Beaverlodge, AB; 55° N, 119° W).^26^ Altogether, this area represents 98.6% of the canola production in Canada and 92.6% in North America.^27^, ^28^ Of these sampled ecoregions, the Peace Lowland region was dominated by *P. striolata* whereas *P. cruciferae* predominated in the Lake Manitoba Plain and Moist Mixed Grasslands based on a study completed in 2011.^29^

We conducted 15 small‐plot canola trials on experimental research stations: (i) Beaverlodge, Alberta (BL) (three trials, *n* = 132 plots), (ii) Lethbridge, Alberta (LB) (three trials, *n* = 120 plots), (iii) Carman, Manitoba (MB) (six trials, *n* = 264 plots), and (iv) Saskatoon, Saskatchewan (SK) (three trials, *n* = 144 plots) (see Supporting Information, Table S1 for details). The trials were completed in 2018, 2019, 2020 (LB); 2018, 2019, 2021 (MB, BL); and 2019, 2021 (SK). All study sites except Lethbridge had to postpone the trials from 2020 to 2021 due to COVID 19 limitations. Plots were planted between mid‐May to early June and ranged from 5 × 1.5 m to 3.6 × 10 m among trials (see Supporting Information, Table S1 for details). The seeding rates used were either a low seeding rate (3.5 kg/ha) to target a plant density of 32.3 plants/m^2^ (three plants/square foot), medium seeding rate (7 kg/ha) to target a plant density of 64.6 plants/m^2^ (six plants/square foot), or a high seeding rate (14 kg/ha) to target a plant density of 129.2 plants/m^2^ (12 plants/square foot). The Canola Council of Canada currently recommends a targeted plant density of 50–80 plants/m^2^ (approximately five to eight plants/square foot),^30^ which would require a seeding rate of approximately 6.5–10.4 kg/ha assuming 60% plant emergence. At each plant density, there were the following flea beetle management treatments: (i) control plots with fungicide only treated seeds (i.e. control) (*n* = 180 plots), (ii) seed treatment with insecticide and fungicide treated seeds (i.e. seed) (*n* = 180 plots), (iii) foliar insecticide spray at 25% defoliation with fungicide only treated seeds (i.e. foliar) (*n* = 180 plots), and (iv) weekly foliar insecticide spray with fungicide only treated seeds to achieve low levels of flea beetle defoliation (i.e. ‘flea beetle‐free’ or ‘FB‐free’) (*n* = 120 plots). The treatment ‘FB‐free’ was added in 2019 to estimate the canola yield achieved by zero to very low levels of flea beetle defoliation, but it was not possible to add it in all trials; the Lethbridge site completed the ‘FB‐free’ treatment in 2019 only, and the other sites completed it in 2019 and 2021. Therefore, trials in 2018 (and LB 2020) contained nine treatments randomized in four blocks resulting in four replicates per treatment (36 plots per trial) and in 2019 and 2021 contained 12 treatments randomized in four blocks resulting in four replicates per treatment (48 plots per trial). The seed cultivar used was SY 4135 (mid‐season Roundup Ready® hybrid canola, laboratory germination 99%; Syngenta, Basel, Switzerland) in all years. Fungicide‐only treatments (control plots) contained Vibrance® Flexi at 600 mL/ 100 kg (difenoconazole, 39.5 g/L; mefenoxam, 12 g/L; sedaxane, 8.4 g/L; fludixonil, 3.6 g/L; Syngenta). In 2018, fungicide plus insecticide‐treated seeds contained VISIVIO™ (Co‐Pack of Helix® Vibrance® at 1500 mL/100 kg and Rascendo™ at 400 mL/100 kg) (sulfoxaflor, 500 g/L; thiamethoxam, 269 g/L; difenoconazole, 16 g/L; metalaxyl‐M, 5 g/L; sedaxane, 3.5 g/L; fludioxonil, 1.7 g/L; Syngenta) and Fortenza® at 500 mL/100 kg (cyantraniliprole, 600 g/L; Syngenta). In all other years, fungicide plus insecticide treatments contained Helix® Vibrance® at 1500 mL/100 kg (thiamethoxam, 269 g/L; difenoconazole, 16 g/L; metalaxyl‐M, 5 g/L; sedaxane, 3.5 g/L; fludioxonil, 1.7 g/L; Syngenta), Rascendo™ at 400 mL/ 100 kg (sulfoxaflor, 500 g/L; Syngenta), and Fortenza® at 500 mL/100 kg (cyantraniliprole, 600 g/L; Syngenta). The foliar insecticide spray used for both foliar and ‘FB‐free’ treatments was Decis® 5 EC (deltamethrin, 50 g/L; Bayer CropScience, Leverkusen, Germany) at 24.3 mL/ha for all trials, except for Saskatoon 2019, which used Matador® 120 EC (lambda‐cyhalothrin, 120 g/L; Syngenta) at 13.8 mL/ha. Foliar treatment plots were sprayed on average two times per trial, ranging from zero to four sprays (see Supporting Information, Table S1 for details). ‘FB‐free’ treatment plots were sprayed weekly starting at 50% plant emergence regardless of flea beetle activity until the 2.4 growth stage, typically lasting around 2–3 weeks.

### 2.2 Sampling methods

Each plot contained one yellow sticky card (18 × 14 cm; Alpha Scent Inc., Canby, OR, USA), with adhesive on both sides of the card, placed vertically at approximately 3 cm above the soil surface with metal stakes near the centre of the plot to monitor flea beetle populations starting at 50% plant emergence. Cards were replaced approximately weekly, depending on weather conditions, until the canola reached the four true‐leaf stage (2.4 stage; 2–3 weeks^31^). Plant density was measured by counting the number of canola plants in three permanent 1‐m linear transects at least 1 m from the plot edges at four separate times: (i) 50% canola emergence, (ii) cotyledon stage (1.0 stage), (iii) two true‐leaf stage (2.2 stage), and (iv) immediately before swathing. The average number of plants in a square metre was then calculated and used to compare plant survivorship between treatments.

Flea beetle defoliation was assessed two times a week on 20 plants per plot by visually estimating percentage damage until approximately 50% of the plant stand reached the 2.4 stage. The defoliation was assessed for the plants' two cotyledons and two true leaves, with each cotyledon or leaf assessed individually. Starting 1 m from the plot edge, the first 10 of 20 plants were assessed in a randomly selected row from the front of each plot (i.e., the edge oriented toward the first block when the trial was viewed left to right, with the first plot of each block on the far left), and another 10 consecutive plants in another randomly selected row in the back of the plot (i.e., the opposite edge). The visual ratings consisted of either a 0–10 scale^32^ where 0 = no defoliation and 10 = entirely defoliated (Lethbridge, AB), converted to percent damage before statistical analyses, or directly estimating percent damage where 0% = no defoliation and 100% = entirely defoliated in 10% intervals (MB, SK, and Beaverlodge, AB). In 2020, due to COVID 19 restrictions, canola ratings were completed by assessing photographs of randomly selected rows for each plot in Lethbridge, AB, which provided similar results to in‐person assessments and the defoliation from this trial still fell within the expected ranges.^32^ Canola was inspected for damage from other pests such as aphids and deer. Foliar insecticide applications were restricted to the most susceptible period to flea beetles (i.e., up to the 2.4 stage).

Weed control of the plots consisted of hand weeding when possible. For plots when hand weeding was not possible, herbicide (glyphosate (540 g/L) at 0.33 L/ac) was applied once or twice per site before the 2.6 stage. Canada thistle was present at both Lethbridge sites, so the glyphosate treatment was tank‐mixed with Lontrel™ 360 at 113 mL/ac (clopyralid, 360 g/L; Corteva, Indianapolis, IN, USA) and applied once after the 2.2 stage. At maturity, the plots were swathed and harvested. The seed was cleaned, and the total seed weights were measured for each plot. The final yield for each plot was estimated as a measure of kg/ha based on the total seed weight per plot and water content.

### 2.3 Data analysis

We tested the effects of our treatments on the number of plants per square metre, total flea beetle abundance, flea beetle abundance per plant, defoliation (%) per plant, and yield. The total flea beetle abundance is a sum of the counted *P. striolata* and *P. cruciferae* individuals per sticky card per day to adjust for the varying trapping durations. The two species were combined since they both show the same general trends in their response to the treatments (Supporting Information, Fig. S1 and Supporting Information, Table S2) and the defoliation caused cannot be attributed to a specific species, but rather the combined effect of both species. A measure of flea beetles per plant was determined by dividing the total number of flea beetles per sticky card by the average number of canola plants in a square metre per plot as a proxy for the flea beetle pressure on each plant. Average plant density was measured from the first count of flea beetles until half the plants per plot reached the 2.4 growth stage. This average was used to account for late plant emergence or mortality across dates, and this estimate reflected the targeted plant density in the treatments. Defoliation per plant is an average of the defoliation found on the two cotyledons and the first two true leaves^33^ of 20 randomly chosen plants per plot twice a week. Canola yield was estimated as kg/ha according to the measured seed weight and plot size, and then standardized at 8.5% moisture.

All variables were analysed using linear mixed effects models and Type III ANOVA with block and plot as random effects (‘lmerTest’ and ‘car’ packages in R^34^, ^35^). The independent variables used were region, flea beetle management treatment, plant density, and their two‐ and three‐way interactions. Year and plot size were both used as covariates. All variables besides yield were transformed to meet the ANOVA assumptions as follows: square root transformation (defoliation) and log transformation (plant number, total flea beetle abundance, and flea beetles per plant). To minimize the unwanted variability from plot size, environmental conditions, and any other variability associated with each trial, the data were centred and scaled per trial for all variables examined.^36^ The final models were chosen using a manual backward selection procedure starting with triple interaction terms among the independent variables and continuing with the two‐way non‐significant interaction terms with the highest *P* values until the final model was retained (Table 1). All individual explanatory variables were kept in the final model. All *post hoc* multiple test comparisons were done using estimated marginal means with the Šidák correction for all significant interactions using the ‘emmeans’ package in R.^37^ Unscaled values averaged at the treatment level are shown in Supporting Information, Tables S3–S7.

**Table 1:** Final linear mixed effects models showing retained parameters after a manual backward selection procedure

		Plant number per m^2^		FB per plot (SC)		FB per plant (m^2^)		Defoliation		Yield	
Model parameter	df	*X* ^2^	*P*	*X* ^2^	*P*	*X* ^2^	*P*	*X* ^2^	*P*	*X* ^2^	*P*
(Intercept)	1	76.71	**<0.001**	31.47	**<0.001**	7.32	**0.007**	15.37	**<0.001**	0.46	0.499
Region	3	0.52	0.915	0.06	0.996	0.65	0.884	8.31	**0.040**	3.10	0.377
FB Mgmt.	3	3.43	0.330	53.00	**<0.001**	23.78	**<0.001**	87.31	**<0.001**	4.49	0.213
PD	2	163.50	**<0.001**	66.84	**<0.001**	57.84	**<0.001**	3.57	0.168	48.83	**<0.001**
Year	2	0.11	0.945	4.54	0.209	2.09	0.352	5.41	0.144	0.76	0.858
Region × FB Mgmt.	9	13.74	0.132					50.11	**<0.001**	23.36	**0.005**
Region × PD	6	10.40	0.109					32.56	**<0.001**		
FB Mgmt. × PD	6	2.62	0.855	20.96	**0.002**	16.55	**0.011**	25.11	**<0.001**		
Region × FB Mgmt. × PD	18	50.61	**<0.001**								

Blank cells indicate that parameter was removed from the final model. Significant parameters are bolded. FB, flea beetle; FB Mgmt., flea beetle management treatment; PD, plant density; SC, sticky card.

## 3 RESULTS

### 3.1 Plant number

On average, seeding rates successfully manipulated plant density, with an average (mean ± SE) plant number per square metre of 122.3 ± 3.5 (11.4 ± 0.3 plants/square foot), 66.2 ± 1.6 (6.1 ± 0.1 plants/square foot), and 38.3 ± 1.3 (3.6 ± 0.1 plants/square foot) in the high, medium, and low plant density treatments, respectively, when averaging from emergence to the 2.4 plant stage. When examining the mean numbers of plants per square metre, a three‐way interaction was found between region, flea beetle management, and plant density (Table 1). All treatments per region showed a significant increase in plant number as plant density increased except for the seed treatment in two trials in SK during 2019, where low and medium plant densities resulted in a similar number of plants, which is likely driving the interaction (Supporting Information, Fig. S2 and Supporting Information, Table S8). Other pairwise comparisons resulting from the three‐way interaction were also significant, specifically a lower density in the seed treatment compared to the foliar and ‘FB‐free’ treatments in MB in the low‐density treatment, but not in a consistent way that may have affected our results (Supporting Information, Fig. S2).

### 3.2 Flea beetle abundance

In general, increasing plant density resulted in a higher abundance of flea beetles; on average low plant density plots had 88.3 ± 6.1 flea beetles/sticky card (72.4 ± 5.7 *P. cruciferae*, 17.4 ± 1.5 *P. striolata*), medium plant density plots had 111.5 ± 7.2 flea beetles (89.2 ± 6.3 *P. cruciferae*, 22.5 ± 1.9 *P. striolata*), and high plant density plots had 135.8 ± 9.4 FB (107.9 ± 7.8 *P. cruciferae*, 28.5 ± 2.7 *P. striolata*). The treatment combination with the lowest flea beetle abundance was low plant density with foliar treatment (81.8 ± 11.3) and the greatest abundance was high plant density control (162.7 ± 20.5). Raw means and SE for each plant density, flea beetle management treatment and region are provided in Supporting Information, Table S3. Within flea beetle management treatments, a higher plant density consistently increased flea beetle abundances, but a two‐way interaction between flea beetle management and plant density treatments (Table 1) resulted from seed and ‘FB‐free’ treatments having only moderate increases of flea beetles with increased plant density compared to other treatments (Fig. 1(a)).

**Figure 1:**
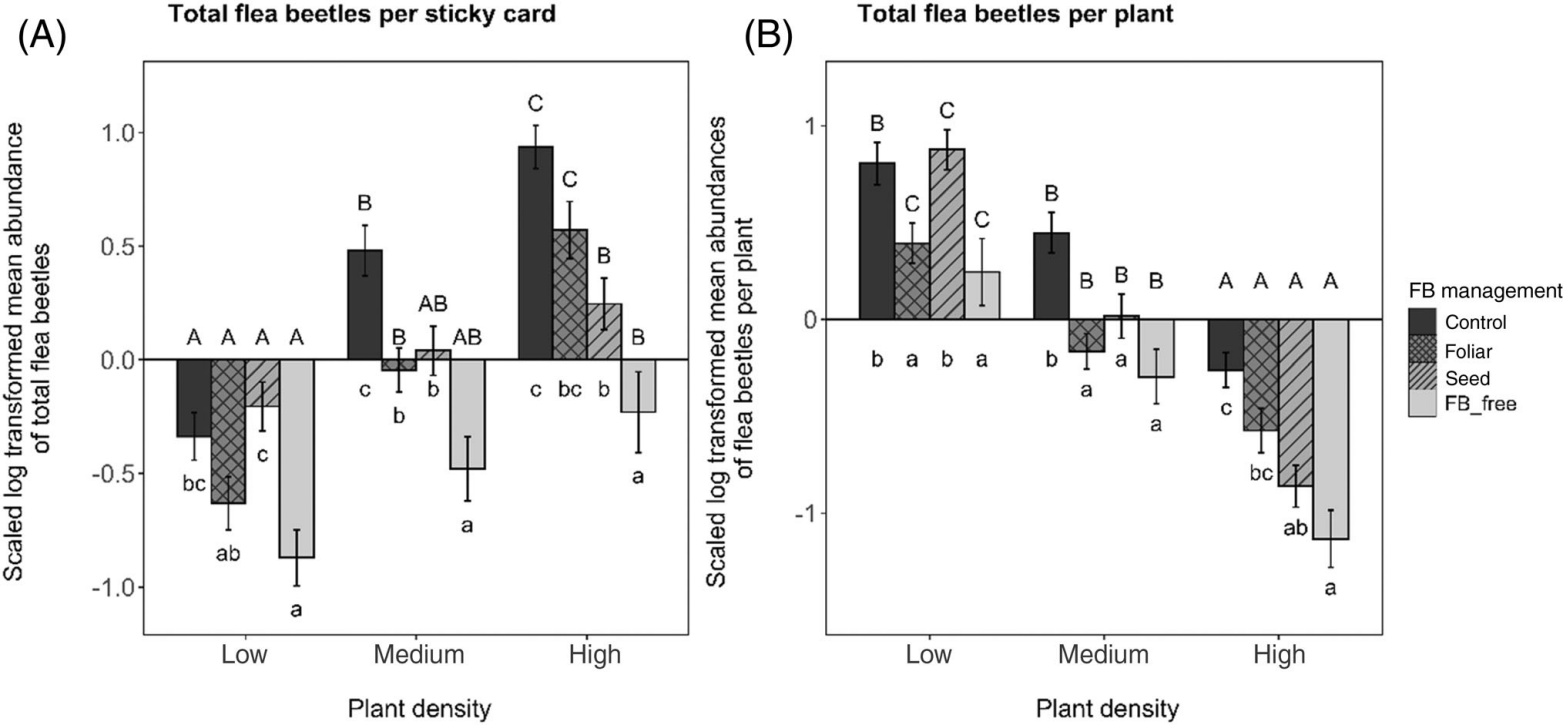
Mean (± SE) scaled log transformed abundances of (A) total flea beetles/sticky trap (*P. cruciferae* + *P. striolata*) based on plant density and flea beetle management treatment for all regions combined and (B) total flea beetles/sticky trap/plant based on plant density and flea beetle management treatment. Capital letters represent differences among plant density treatments within each flea beetle management treatment/region. Lower‐case letters represent differences among flea beetle management treatments/region within each plant density treatment (Estimated marginal means, *⍺* = 0.05).

On a per plant basis, we observed the opposite pattern, with a reduction in FB per plant with plant density increase (Fig. 1(B)). Low plant density plots had 2.6 ± 0.2 FB/sticky trap/plant (1.9 ± 0.2 and 0.7 ± 0.1 for *P. cruciferae* and *P. striolata*, respectively), medium plant density plots had 1.9 ± 0.1 flea beetles/sticky trap/plant (1.4 ± 0.1 and 0.4 ± 0.0 for *P. cruciferae* and *P. striolata*, respectively), and high plant density plots had 1.3 ± 0.1 flea beetles/sticky trap/plant (1.0 ± 0.1 and 0.3 ± 0.0 for *P. cruciferae* and *P. striolata*, respectively). The treatment with the lowest flea beetles/sticky trap/plant was high plant density with seed treatment (1.0 ± 0.2) and the greatest abundance was low plant density with seed treatment (2.9 ± 0.4). Raw means and SE for each plant density, flea beetle management treatment, and region are provided in Supporting Information, Table S5. The significant interaction between plant density and flea beetle management (Table 1) was likely due to the lack of difference in flea beetles per plant between low and medium plant density on the control treatment, and to the seed treatment resulting in the greatest amount of flea beetles per plant at low densities, yet it was grouped with the ‘FB‐free’ treatment as having the lowest number of flea beetles per plant at high plant densities (Fig. 1(B)).

### 3.3 Defoliation

Despite effects of plant density on flea beetle total abundance and abundance per plant, there was no consistent impact of defoliation associated with plant density and flea beetle management treatments. On average, low plant density plots had 20.6 ± 0.9% defoliation, medium plant density plots had 20.6 ± 0.9% defoliation, and high plant density plots had 18.9 ± 0.9% defoliation. The treatment with the lowest defoliation was high plant density with seed treatment (10.3 ± 0.7%) and the greatest defoliation was medium plant density control (29.8 ± 2.0%). Raw means and SE for each plant density, flea beetle management treatment and region are provided in Supporting Information, Table S6. All two‐way interactions were significant (Table 1 and Fig. 2). Defoliation was higher in the control and foliar spray treatments compared with the seed and flea beetle free treatments at all plant density (Fig. 2(A)) and regions (Fig. 2(B)). Defoliation levels were generally similar within flea beetle management treatments except for lower defoliation at high plant density within the seed treatment (Fig. 2(A)). The interaction between region and FB management was likely due to the lowest defoliation in the seed treatment but the highest defoliation in the FB treatment in Saskatoon compared to other regions, whereas regions did not differ in defoliation in control and foliar treatments (Fig. 2(B)). The interaction between region and plant density was probably due to defoliation in Saskatoon and Carman decreasing with plant density increases contrasting with the opposite pattern observed for Lethbridge and the lack of differences observed in Beaverlodge (Fig. 2(C)).

**Figure 2:**
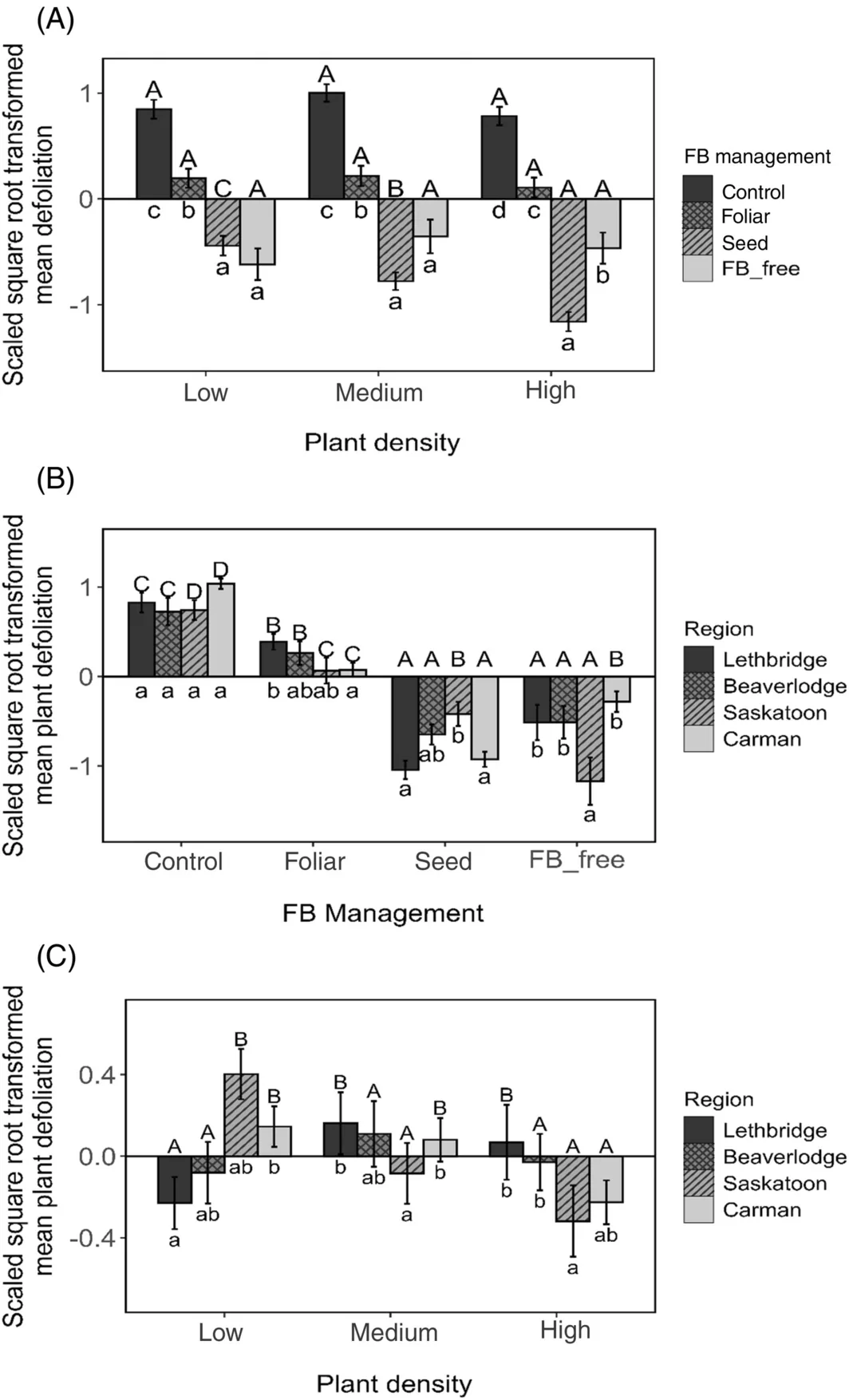
Mean (± SE) scaled square root transformed percentages of defoliation per plot based on (A) plant density and flea beetle management treatment for all regions combined, (B) plant density for each region, and (C) flea beetle management treatment for each region. Capital letters represent differences among (A, B) plant density treatments or (C) flea beetle management treatment within each (A) flea beetle management treatment or (B, C) region. Lower‐case letters represent differences among (A) flea beetle management treatments or (B, C) region within each (A, B) plant density treatment or (C) flea beetle management treatment (Estimated marginal means, *⍺* = 0.05).

### 3.4 Yield

Yield was significantly increased by plant density across all flea beetle management treatments and regions (Table 1 and Fig. 3(A)). On average, low plant density plots had a yield of 1321 ± 79.0 kg/ha, medium plant density plots had 1535.5 ± 86.9 kg/ha, and high plant density plots had 1588.7 ± 85.6 kg/ha. Raw means and SE for each plant density, flea beetle management treatment and region are provided in Supporting Information, Table S7. Independently of plant density, the yield was significantly affected by an interaction between region and flea beetle management treatment (Table 1). All regions showed different trends when considering which flea beetle management treatment results in a higher yield (Fig. 3(B)). Beaverlodge shows no differences in yield across any management strategy, while other regions show a yield increase compared to the control plots with the seed treatment (Lethbridge), foliar and seed treatment (Carman), or the ‘FB‐free’ treatment (Carman and Saskatoon) (Fig. 3(B)). Within flea beetle management treatments, regions did not have significant effects (Fig. 3(B)).

**Figure 3:**
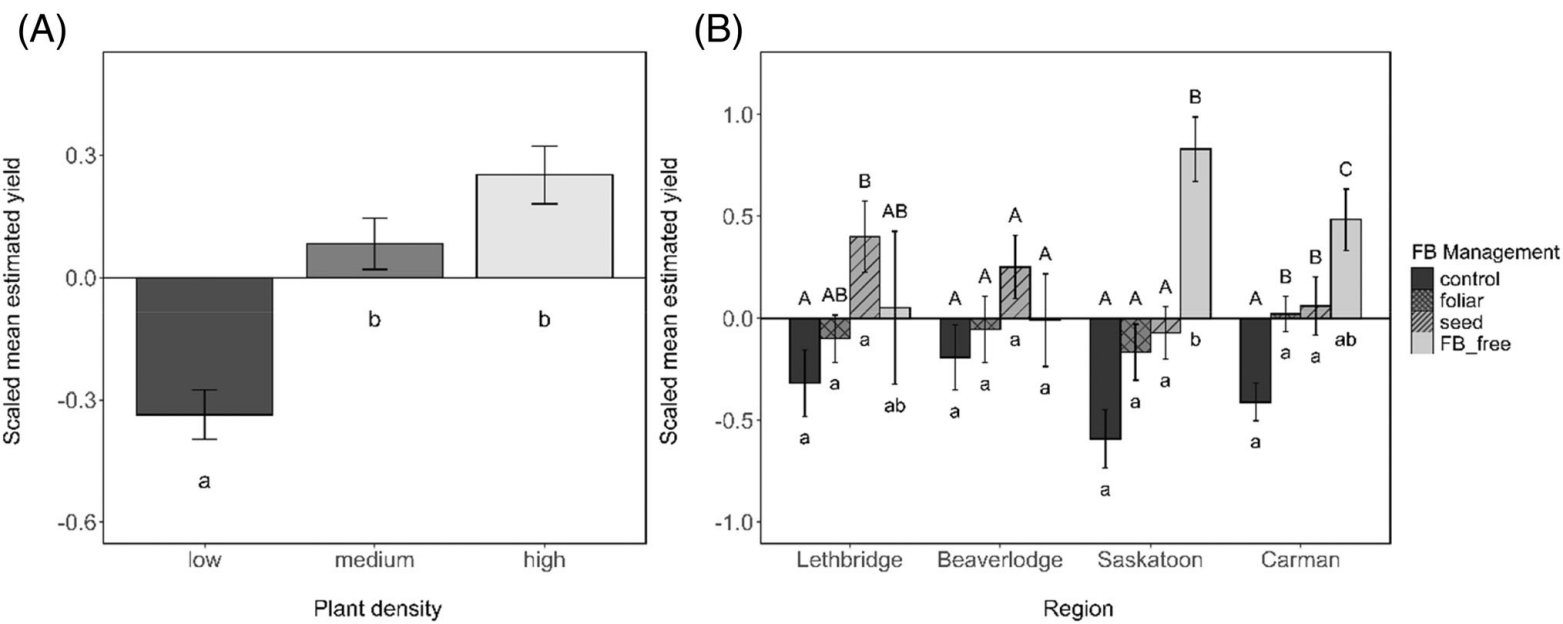
Mean (± SE) scaled yield per plot based on (A) plant density and (B) flea beetle management treatment for each region. Yield is estimated and corrected at 8.5% moisture. Capital letters represent differences among flea beetle management treatment within each region. Lower‐case letters represent differences among regions within each flea beetle management treatment (Estimated marginal means, *⍺* = 0.05). Within flea beetle management treatments, regions did not significantly differ.

## 4 DISCUSSION

Our results show that flea beetles were more abundant in plots with a higher canola density when compared to more sparse plots, supporting the resource concentration hypothesis.^38^ Comparing pure or dense stands of collards with rows of collards in diverse mixed plant stands, Root^38^ observed that specialist herbivores, including *P. cruciferae*, were found at higher abundance in pure than in diverse stands of collard plants. Further studies manipulating collard patch sizes suggested that increasing fragmentation and reducing patch size also reduce flea beetle abundance.^39^, ^40^ Flea beetles are likely to aggregate and immigrate into *Brassica*‐dense areas and move from small patches to large patches based on the increase in volatiles that *Brassica* plants produce,^41^, ^42^ and are also more likely to emigrate from smaller patches.^41^ Although these studies were conducted at small, plot‐level scales, the same process of flea beetle aggregation and dispersal can operate at broader landscape‐level scales where fields can vary in size and spatial isolation. Recently, studies conducted at landscape scales in Sweden, where fields are on average smaller than in the Canadian prairies (mean field size = 17.56 ± 2.35 ha), suggest that spatial isolation of the previous season's canola fields by 5 km decrease flea beetle abundance and defoliation enough to increase yield.^20^ These results indicate that aggregation and retention of flea beetles may increase at higher plant densities, yet we demonstrate that the increased density does not translate into increased defoliation and is offset by the higher yield observed at both medium and high plant densities compared to the low‐density plots. Further studies should investigate these patterns in large field scales to design scouting and management techniques appropriate for commercial fields in North America and Europe.

Canola plants in high‐density plots experienced lower flea beetle pressure on a per plant basis, counteracting the aggregation of flea beetles and resulting in an overall dilution effect of increased plant density. Previous research manipulating plant density in Alberta, Canada, and Sweden also suggested dilution effects on canola based on a reduction of defoliation at increased plant densities under a variety of conditions, including canola species, different seed treatments, planting dates,^22^, ^43^ row spacing, and tillage regimes^23^; however, most studies did not quantify flea beetle abundance. Dosdall *et al*.^22^ report that increasing seeding rates to 12.5 kg/ha in untreated plots reduced canola damage to similar levels as lower seeding rates treated with seed insecticides, suggesting that increasing plant density can contribute to managing flea beetles without prophylactic applications of insecticides in seeds. Here, we expand previous results by demonstrating dilution effects due to a reduction on flea beetle density per plant and increase yield in multiple trials across a wide region and under two flea beetle management regimes: seed‐coated neonicotinoids and foliar pyrethroid treatments.

Interestingly, the defoliation levels per plant in this study did not show a clear dilution effect the same way the flea beetles per plant did. Increased plant density reduced defoliation only in the seed treatment but not in the other flea beetle management treatments. The control treatment, which lacks insecticide, shows that even when flea beetle abundances are higher per plant in low density plots, the defoliation did not increase in a predictable way. Rather, it shows no difference from the defoliation observed in other plant density treatments. Similar results have been reported in other oilseed systems, for example studies on *Psylliodes* flea beetles showed that increased plant density did not consistently reduce damage.^44^ An explanation for the lack of trend observed is that flea beetles are increasing their searching time in low density plots due to the larger distances between plants, which in turn results in less feeding time. Another management strategy that may decrease oilseed pest damage such as *Phyllotreta*, *Psylliodes*, and *Brassicogethes* is undersowing companion plants, which may also increase searching time without raising canola seed costs.^9^, ^45^, ^46^ Additionally, collards that were planted alongside crucifer weeds resulted in less damage per plant.^47^ However, in western Canada, intercropping canola with wheat did not result in consistent crop protection against flea beetles, thus it was not recommended as a pest management strategy for wide adoption.^48^

Regionally, plant density reduced defoliation in Carman and Saskatoon, but it showed the reverse trend in Lethbridge and, under lower defoliation levels, showed no effect on Beaverlodge. The lack of significant differences between treatments in Beaverlodge specifically could also be explained by the low numbers of flea beetles captured per plot combined with the large plot size (Supporting Information, Table S3). In addition, the relative abundance of *P. cruciferae* and *P. striolata* varied across the Canadian prairies. In our study, *P. cruciferae* was found in greater numbers than *P. striolata*, except for Saskatoon in 2019 and the Beaverlodge region, where the numbers of *P. cruciferae* were approaching zero and there were also relatively few *P. striolata* (Supporting Information, Table S4), which supports previous reports.^29^ Soroka *et al*.^29^ reported that *P. striolata* comprised 98% of the flea beetle population in Beaverlodge and 89% in Saskatoon in 2011. Our results show a similar pattern in Beaverlodge, where *P. striolata* made up 92.8–100.0% of the population between 2018 and 2021. In Saskatoon, however, *P. striolata* were less dominant: they comprised 65.9% of the population in 2019 and only 43.1% in 2021, suggesting a recent shift towards *P. cruciferae* as the dominant species. In summary, these results combined with previous research suggest that increasing plant density results in dilution effects that reduce flea beetle pressure per plant, which in turn may reduce defoliation under certain conditions. Further studies are needed to establish the factors governing flea beetle damage under field conditions.

The defoliation levels observed in our study are consistent with previous findings in the Canadian prairie provinces. Dosdall *et al*.^22^ showed mean flea beetle defoliation on *B. napus* ranging from 5% to 27% defoliation. Similarly, Cárcamo *et al*.^49^ found that flea beetle defoliation in Alberta ranged from 0% to 36% while Dosdall and Stevenson^23^ reported 9.9% as being their highest levels of defoliation across their sites in Alberta. This research shows mean defoliation in untreated controls ranging from as little as 8.5% in Beaverlodge (2021) to 53.3% in Carman (2019), with approximately 1/4 of the plots exceeding the 25% economic threshold throughout the entire study. Interestingly, previous results combined with the new data from small plots conducted in multiple regions across western Canada and across 4 years show that typical flea beetle defoliation levels fall below the 25% threshold damage. Our canola seed yield results were within the range of yield obtained in previous field experiments in the regions where these trials were conducted.^50^, ^51^

As expected, flea beetle management treatments affected flea beetle defoliation, with either seed or ‘FB‐free’ (weekly pyrethroid foliar application) treatments being the most effective at reducing the defoliation when compared to the control depending on the region or plant density. In terms of management regimes, seed‐coated treatments are already the main strategy used across the Canadian prairies.^51^, ^52^ These results confirm previous reports on the effectiveness of using neonicotinoid‐coated seeds to reduce defoliation by flea beetles.^53^, ^54^, ^55^ The reduction of defoliation observed at higher plant density in the seed treatment plots may be due to the increase in insecticide product in the whole plot because there were more seeds (and therefore insecticide) at higher plant density. However, the exact mechanism of this effect remains to be investigated. Interestingly, 43 of the 45 seed‐by‐plan‐density treatment combinations across all trials successfully maintained flea beetle defoliation below the 25% nominal threshold. The two instances where the seed treatment exceeded 25% damage occurred only in the low‐density plots. The success of the seed treatment in these trials suggests that follow‐up foliar treatment applications that are common across the prairies may not be necessary in many cases. Despite their effectiveness, the use of neonicotinoids may lead to unintended and detrimental effects in terms of reduced effectiveness towards striped flea beetles due to tolerance,^56^ pest resurgence,^53^ and impacts on non‐target organisms,^10^ so alternative methods of control should be considered to reduce the current reliance on this control method. The motivation to adopt such alternative methods may also vary depending on local flea beetle pressure. For example, Beaverlodge consistently showed low flea beetle abundance in our trials, which raises questions about whether seed treatments provide the same relative benefit in this region compared to areas with higher pest pressure. Finally, although the foliar spray treatment at the 25% defoliation threshold generally resulted in slightly less defoliation reduction than the seed treatment, it always reduced defoliation compared to controls. The advantage of this method is that it results in insecticide applications only in response to economic populations of flea beetles, reducing unnecessary prophylactic applications of systemic insecticides on seed.^17^ Relying solely on this method requires more active, ideally daily, scouting of canola fields to ensure timely application of control measures when needed, which is not always practical for growers.^57^ However, foliar sprays can also pose challenges, including added logistical demands for canola growers during a busy time of the season and a higher risk of applicator exposure and health complications.^58^ Therefore, our results suggest that developing cost‐effective scouting techniques in canola fields (e.g., using drones, developing real‐time AI optical damage assessment tools, forecasting models, smart traps, etc.^9^, ^59^, ^60^) will contribute to reduce unnecessary foliar sprays that will not result in yield increase.

Increased plant density resulted in higher canola yield, independently of flea beetle abundance, species composition, region, or flea beetle management strategy over a wide region of western Canada during the 4 years of this study. Increasing plant density is compatible with the management of other insect pests in canola^22^, ^23^ and other techniques that may further reduce flea beetle defoliation, including no‐till, manipulation of row spacing, larger seed sizes, varying planting date and ratios of insecticide‐treated and untreated seeds.^2^, ^22^, ^23^, ^43^, ^49^, ^51^ However, the economic benefit from an increased yield would not outweigh the high cost of the seed at 14 kg/ha. Increasing the plant density by 10 plants/m^2^ is likely not economical due to the high seed cost compared to low foliar insecticide application costs,^9^ which implies that our suggested plant density increase from low to medium of approximately 30 plants/m^2^ would be far too costly for Canadian canola producers. Establishing high‐density trap crop^61^, ^62^, ^63^ strips along the margins of canola fields to attract flea beetles away from the main crop could be a useful compromise and a viable management strategy to protect yield while keeping seed costs lower, warranting further investigation. However, there are documented cases where very high seeding rates (e.g., >150 plants/m^2^) can lead to yield declines either due to competition, lodging, or accelerated maturity resulting in pod shattering.^64^, ^65^ In addition, future research should examine the actual and estimated costs of seed treatment and foliar insecticides to conduct a cost–benefit analysis of higher seeding rates, assessing whether the yield gains and reduced pesticide costs offset the added seed expense. Nevertheless, our research showed that despite the instances where the seed treatment does exceed the 25% damage threshold across four regions and 3 years, yield was primarily driven by plant density regardless of trial, region, or management strategy.

## 5 CONCLUSION

The current study shows that increased canola density resulted in an overall increase in yield and a decrease in flea beetle abundance per plant, offsetting a higher abundance of flea beetles observed in the moderate and high plant density treatments. This general trend was consistent among the different regions, flea beetle species assemblages, and flea beetle management treatments and suggests that higher plant density increases canola yield and reduces the impact of flea beetles in the Canadian prairies. Our trials were conducted over a range of representative but variable conditions of soil, temperature, and precipitation, and on small‐size replicated plots with unbalanced representation across the regions. Thus, although this variability was accounted for by centring and scaling the data, results should be interpreted with some caution before extrapolating to other environmental conditions, commercial field sizes, and regions. Increasing plant density in combination with other management strategies can provide a more sustainable method to manage flea beetle populations and reduce reliance on prophylactic treatments of seeds with insecticides.^9^, ^22^, ^66^

## FUNDING INFORMATION

This work was supported by Canola AgriScience Cluster Program (Project ASC‐02). SW received research funding from a James Gordon Fletcher graduate fellowship and the University of Manitoba.

## CONFLICT OF INTEREST

The authors have no relevant financial or non‐financial interests to disclose.

## AUTHOR CONTRIBUTIONS

Hector A. Cárcamo, Tyler Wist, Jennifer Otani, John Gavloski, Robert W. Dunan, Tharshinidevy Nagalingam, and Alejandro C. Costamagna contributed to the conceptualization of the study design and funding acquisition. Data collection and material preparation were performed by all authors. Data was curated and analyzed by Shayla Woodland. The first draft of the manuscript was written by Shayla Woodland and all authors commented on previous versions of the manuscript. All authors read and approved the final manuscript. Conceptualization: HC, TW, JO, JG, RD, TN, and ACC. Data curation: SW. Formal analysis: SW. Funding acquisition: HC, TW, JO, JG, RD, TN, and ACC. Investigation: SW, HC, TW, JO, JG, RD, and ACC. Methodology: SW, HC, TW, JO, JG, RD, TN, and ACC. Project administration: HC, TW, JO, JG, RD, TN, and ACC. Resources: HC, TW, JO, JG, RD, TN, and ACC. Supervision: HC, TW, JO, JG, RD, TN, and ACC. Writing – original draft: SW. Writing – review and editing: SW, HC, TW, JO, JG, RD, TN, and ACC.

## Supporting information


**Table S1.** Details for canola study sites used to determine the effects of flea beetle management strategies and plant density treatments on flea beetle populations, defoliation, and canola yield.
**Table S2.** Additional GLMM models showing retained parameters after a manual backward selection procedure for SFB and CFB per sticky card (SC) (per plot). Blank cells indicate that parameter was removed from the final model. Significant parameters are bolded. FB Mgtmt., flea beetle management treatment; PD, plant density.
**Table S3.** Mean (± SE) flea beetles (FB) per sticky card (per plot) based on collection date among 3 years, three plant densities, and four FB management treatments.
**Table S4.** Mean (± SE) flea beetles (FB) per sticky card (per plot) based on species, region, and year collected.
**Table S5.** Mean (± SE) flea beetles (FB) per sticky card per plant based on plot among 3 years, three plant densities, and four FB management treatments.
**Table S6.** Mean (± SE) defoliation (whole plant) (%) per plot among 3 years, three plant densities, and four flea beetle (FB) management treatments.
**Table S7.** Mean (± SE) yield (corrected at 8.5% moisture) (kg/ha) per plot among 3 years, three plant densities, and four flea beetle (FB) management treatments
**Table S8.** Mean (± SE) plants per square metre, yield (corrected at 8.5% moisture) (kg/ha), and defoliation (%) per trial in each region among 3 years, three plant densities, and four flea beetle (FB) management treatments
**Figure S1.** Mean (± SE) scaled log transformed abundances of (A) total SFB/sticky card and (B) total CFB/sticky card based on PD and FB management treatment for all regions combined. Capital letters represent differences among PD treatments within each FB management treatment/region. Lower‐case letters represent differences among FB management treatments/region within each PD treatment (EMM, *⍺* = 0.05). FB, flea beetle; PD, plant density; SFB, striped flea beetle; CFB, crucifer flea beetle.

## Data Availability

The data that support the findings of this study are available from the corresponding author upon reasonable request.
