# Supplementary material for: Combined effects of canola plant density and insecticide management strategies on flea beetle abundance, canola defoliation, and yield across the Canadian prairies
Source: Pest Manag Sci. 2025 Oct 30;82(11):10648–57. doi: 10.1002/ps.70320 (PMC13665633; doi:10.1002/ps.70320)
Supplement: Supplementary file 1 [file PS-82-10648-s001.docx]

##### **Table S1.** Details for canola study sites used to determine the effects of flea beetle management strategies and plant density treatments on flea beetle populations, defoliation, and canola yield.

| **Year** | **Province** | | **Site** ^†^ | **Plot Size (m)** | **Row**  **Spacing (cm)** | | **Seeded Date** | **Times Sprayed Foliar** | **Dates Sprayed Foliar** | **Flea Beetle-free Control** |
| --- | --- | --- | --- | --- | --- | --- | --- | --- | --- | --- |
| 2018 | AB ^‡^ | Lethbridge | | 2.9 x 8 | 24 | 13-May-18 | | 2 | 29-May-18 04-Jun-18 | no |
|  | AB | Beaverlodge | | 3.6 x 10 | 23 | 19-May-18 | | 2 | 05-Jun-18 | no |
|  |  |  |  |  |  |  |  |  | 09-Jun-18 |  |
|  | MB | Carman 1 | | 1.5 x 5 | 23 | 11-May-18 | | 2 | 28-May-18 | no |
|  |  |  |  |  |  |  |  |  | 04-Jun-18 |  |
|  | MB | Carman 2 | | 1.5 x 5 | 23 | 11-May-18 | | 2 | 28-May-18 | no |
|  |  |  |  |  |  |  |  |  | 04-Jun-18 |  |
| 2019 | AB | Lethbridge | | 2.9 x 8 | 24 | 11-May-19 | | 3 | 21-May-19 | yes |
|  |  |  |  |  |  |  |  |  | 05-Jun-19 |  |
|  |  |  |  |  |  |  |  |  | 12-Jun-19 |  |
|  | AB | Beaverlodge | | 3.6 x 10 | 23 | 11-May-19 | | 2 | 31-May-19 | yes |
|  |  |  |  |  |  |  |  |  | 05-Jun-19 |  |
|  | SK | Saskatoon 1 | | 1.5 x 6.1 | 23 | 11-May-19 | | 4 | 05-Jun-19  06-Jun-19  08-Jun-19  19-Jun-19 | yes |
|  | SK | Saskatoon 2 | | 1.5 x 6.1 | 23 | 06-Jun-19 | | 2 | 19-Jun-19  04-Jul-19 | yes |
|  | MB | Carman 1 | | 1.5 x 5 | 23 | 14-May-19 | | 4 | 30-May-19 | yes |
|  |  |  | |  |  |  | |  | 04-Jun-19 |  |
|  |  |  | |  |  |  | |  | 06-Jun-19 |  |
|  |  |  | |  |  |  | |  | 14-Jun-19 |  |
|  | MB | Carman 2 | | 1.5 x 5 | 23 | 14-May-19 | | 4 | 30-May-19 | yes |
|  |  |  | |  |  |  | |  | 04-Jun-19 |  |
|  |  |  | |  |  |  | |  | 10-Jun-19 |  |
|  |  |  | |  |  |  | |  | 14-Jun-19 |  |
| 2020 | AB | Lethbridge | | 2.9 x 8 | 24 | 12-May-20 | | 3 | 04-Jun-20 | no |
|  |  |  |  |  |  |  |  |  | 11-Jun-20 |  |
|  |  |  |  |  |  |  |  |  | 17-Jun-20 |  |
| 2021 | AB | Beaverlodge | | 3.6 x 10 | 23 | 11-May-21 | | 1 | 10-Jun-21 | yes^§^ |
|  | SK | Saskatoon | | 1.5 x 6.1 | 23 | 31-May-21 | | 3 | 07-Jun-21  15-Jun-21  18-Jun-21 | yes |
|  | MB | Carman 1 | | 1.5 x 5 | 23 | 13-May-21 | | 3 | 03-Jun-21 | yes |
|  |  |  |  |  |  |  |  |  | 10-Jun-21 |  |
|  |  |  |  |  |  |  |  |  | 17-Jun-21 |  |
|  | MB | Carman 2 | | 1.5 x 5 | 23 | 31-May-21 | | 0^¶^ | NA | yes |

^†^ Plots were planted either 1.0 m (LB), 2.0 m (BL), 1.5 m (MB), or 2.4 m (SK) apart. SK plots each had 2 rows of barley as guards on each side, all other locations had empty rows. In BL, each block was spaced 15 m apart and was seeded to fall rye in early June (9-inch row spacing). Each block in BL also had one buffer plot of canola (*B.* napus cv. 74-54-RR at 6 kg.ha) on either end to control for edge effects. Seeding information (all on heavy disced soil in the spring): LB = Pillar laser disc-hoe openers, air drill seeding; BL = ConservaPak seeder; MB = Wintersteiger seeder with double disc openers, SK = Hege 4-row cone-belt seeder.

^‡^ LB = Lethbridge, Alberta; BL= Beaverlodge, Alberta; MB = Carman, Manitoba; SK = Saskatoon, Saskatchewan

^§^ Flea beetle-free control treatment only sprayed once due to COVID field limitations
^¶^ Foliar treatment plots never reached 20-25% injury damage threshold.

**Table S2.** Additional GLMM models showing retained parameters after a manual backward selection procedure for SFB and CFB per sticky card (SC) (per plot). Blank cells indicate that parameter was removed from the final model. Significant parameters are bolded. FB Mgtmt. = flea beetle management treatment; PD = plant density.

|  |  | **SFB per plot (SC)** | | **CFB per plot (SC)** | |
| --- | --- | --- | --- | --- | --- |
| **Model parameter** | **DF** | **X^2^** | ***p*** | **X^2^** | ***p*** |
| (Intercept) | 1 | 29.91 | **<0.001** | 25.28 | **<0.001** |
| Region | 3 | 2.16 | 0.540 | 0.01 | 1.000 |
| FB Mgmt. | 3 | 50.91 | **<0.001** | 46.89 | **<0.001** |
| PD | 2 | 44.64 | **<0.001** | 64.77 | **<0.001** |
| Year | 2 | 2.81 | 0.421 | 2.75 | 0.432 |
| Region x FB Mgmt. | 9 |  |  |  |  |
| Region x PD | 6 | 14.37 | **0.026** |  |  |
| FB Mgmt x PD | 6 | 15.48 | **0.017** | 31.61 | **<0.001** |
| Region x FB Mgmt. x PD | 18 |  |  |  |  |

**Table S3**. Mean (± SE) flea beetles (FB) per sticky card (per plot) based on collection date among 3 years, 3 plant densities, and 4 FB management treatments.

| **Year** | **Plant density** | **FB Management** | **LB^†^** | | | **BL** | | | **MB** | | | **SK** | | | |  |
| --- | --- | --- | --- | --- | --- | --- | --- | --- | --- | --- | --- | --- | --- | --- | --- | --- |
| **2018** | Low | Control | 83.6 | ± | 15.5 | 7.2 | ± | 1.1 | 52.9 | ± | 6.8 | NA* | | | |  |
|  |  | Foliar | 86.8 | ± | 24.3 | 5.4 | ± | 0.7 | 56.5 | ± | 8.0 |  |  |  |  |  |
|  |  | Seed | 75.8 | ± | 4.7 | 7.8 | ± | 0.3 | 61.5 | ± | 6.6 |  |  |  |  |  |
|  | Optimum | Control | 211.7 | ± | 56.9 | 11.0 | ± | 1.7 | 96.4 | ± | 16.3 | NA | | | |  |
|  |  | Foliar | 235.3 | ± | 48.1 | 7.4 | ± | 0.7 | 75.3 | ± | 10.9 |  |  |  |  |  |
|  |  | Seed | 106.5 | ± | 12.3 | 8.9 | ± | 1.3 | 57.5 | ± | 4.4 |  |  |  |  |  |
|  | High | Control | 452.0 | ± | 73.3 | 14.6 | ± | 1.7 | 141.9 | ± | 17.2 | NA | | | |  |
|  |  | Foliar | 447.2 | ± | 44.2 | 9.9 | ± | 2.7 | 126.1 | ± | 19.8 |  |  |  |  |  |
|  |  | Seed | 98.3 | ± | 13.2 | 12.3 | ± | 2.8 | 67.2 | ± | 7.3 |  |  |  |  |  |
| **FB averages 2018** | | | **199.7** | **±** | **27.0** | **9.4** | **±** | **0.7** | **81.7** | **±** | **5.3** |  | **-** | |  | |
| **2019** | Low | Control | 246.4 | ± | 15.4 | 22.0 | ± | 5.8 | 192.1 | ± | 37.4 | 34.3 | ± | 9.5 | |  |
|  |  | Foliar | 241.3 | ± | 10.1 | 22.3 | ± | 1.2 | 148.0 | ± | 32.8 | 27.4 | ± | 5.4 | |  |
|  |  | Seed | 307.6 | ± | 5.1 | 14.4 | ± | 2.7 | 152.8 | ± | 27.6 | 47.4 | ± | 13.8 | |  |
|  |  | FB-Free | 217.6 | ± | 8.2 | 16.8 | ± | 3.4 | 144.9 | ± | 26.2 | 26.1 | ± | 8.5 | |  |
|  | Optimum | Control | 352.4 | ± | 23.1 | 55.1 | ± | 31.9 | 278.7 | ± | 38.3 | 49.0 | ± | 15.0 | |  |
|  |  | Foliar | 222.1 | ± | 19.3 | 26.8 | ± | 1.7 | 211.8 | ± | 42.6 | 46.5 | ± | 13.5 | |  |
|  |  | Seed | 318.5 | ± | 22.9 | 17.0 | ± | 2.5 | 209.7 | ± | 39.4 | 31.2 | ± | 8.3 | |  |
|  |  | FB-Free | 228.9 | ± | 22.9 | 26.5 | ± | 7.6 | 169.3 | ± | 26.5 | 30.9 | ± | 10.8 | |  |
|  | High | Control | 368.7 | ± | 42.2 | 31.6 | ± | 2.8 | 331.4 | ± | 60.5 | 65.1 | ± | 18.4 | |  |
|  |  | Foliar | 300.0 | ± | 36.4 | 34.3 | ± | 3.7 | 260.6 | ± | 44.1 | 56.9 | ± | 16.1 | |  |
|  |  | Seed | 280.7 | ± | 28.7 | 23.0 | ± | 3.1 | 204.6 | ± | 42.1 | 42.8 | ± | 11.8 | |  |
|  |  | FB-Free | 305.8 | ± | 38.3 | 24.8 | ± | 1.9 | 287.2 | ± | 74.0 | 28.9 | ± | 10.2 | |  |
| **FB averages 2019** | | | **282.5** | **±** | **9.6** | **26.2** | **±** | **2.9** | **215.9** | **±** | **13.2** | **40.5** | **±** | **3.6** | |  |
| **2020/2021** ^§^ | Low | Control | 74.9 | ± | 8.8 | 3.6 | ± | 0.7 | 179.6 | ± | 28.3 | 18.9 | ± | 4.0 | |  |
|  |  | Foliar | 71.9 | ± | 19.2 | 2.1 | ± | 0.6 | 156.9 | ± | 38.6 | 19.4 | ± | 3.4 | |  |
|  |  | Seed | 125.4 | ± | 21.5 | 1.9 | ± | 0.2 | 176.3 | ± | 32.0 | 21.9 | ± | 1.8 | |  |
|  |  | FB-Free | NA | | | 1.3 | ± | 0.3 | 111.3 | ± | 23.1 | 24.4 | ± | 4.4 | |  |
|  | Optimum | Control | 118.7 | ± | 10.4 | 3.8 | ± | 0.5 | 182.0 | ± | 27.1 | 23.8 | ± | 1.3 | |  |
|  |  | Foliar | 105.5 | ± | 7.1 | 2.7 | ± | 0.5 | 142.5 | ± | 19.2 | 22.6 | ± | 4.7 | |  |
|  |  | Seed | 157.6 | ± | 10.4 | 4.1 | ± | 0.7 | 205.6 | ± | 27.0 | 23.1 | ± | 3.5 | |  |
|  |  | FB-Free | NA | | | 4.8 | ± | 0.6 | 125.0 | ± | 17.7 | 14.8 | ± | 3.2 | |  |
|  | High | Control | 101.7 | ± | 5.6 | 6.6 | ± | 1.3 | 175.4 | ± | 18.7 | 38.5 | ± | 10.1 | |  |
|  |  | Foliar | 71.5 | ± | 20.5 | 7.0 | ± | 1.4 | 170.5 | ± | 25.8 | 39.5 | ± | 5.8 | |  |
|  |  | Seed | 135.6 | ± | 30.4 | 6.6 | ± | 1.6 | 222.7 | ± | 23.8 | 26.5 | ± | 3.7 | |  |
|  |  | FB-Free | NA | | | 3.1 | ± | 0.6 | 114.6 | ± | 17.6 | 30.9 | ± | 15.6 | |  |
| **FB averages 2020/2021** | | | **107.0** | **±** | **6.9** | **4.0** | **±** | **0.3** | **163.5** | **±** | **7.8** | **25.3** | **±** | **1.9** | |  |

**^†^**LB = Lethbridge, Alberta; BL= Beaverlodge, Alberta; MB = Carman, Manitoba; SK = Saskatoon, Saskatchewan

^‡^ NAs indicate data missing due to inability to access fields, collect data, or lack of personnel due to either COVID-19 limitations or other reasons preventing proper protocol

^§^ 2020: Lethbridge (AB); 2021, Beaverlodge (AB), MB, SK – different years due to different COVID-19 limitations

**Table S4.** Mean (± SE) flea beetles (FB) per sticky card (per plot) based on species, region and year collected.

| **Year** | **FB species** | **LB^†^** | | | **BL** | | | **MB** | | | **SK** | | |
| --- | --- | --- | --- | --- | --- | --- | --- | --- | --- | --- | --- | --- | --- |
| **2018** | SFB ^‡^ | 30.1 | ± | 4.8 | 8.5 | ± | 0.5 | 13.9 | ± | 1.0 | NA | | |
|  | CFB | 169.9 | ± | 22.4 | 0.9 | ± | 0.2 | 67.8 | ± | 4.3 |  |  |  |
| **2019** | SFB | 25.8 | ± | 0.9 | 26.2 | ± | 2.9 | 65.3 | ± | 5.7 | 27.1 | ± | 2.3 |
|  | CFB | 256.6 | ± | 9.1 | 0.0 | ± | 0.0 | 150.6 | ± | 8.1 | 13.4 | ± | 1.4 |
| **2020/2021** ^§^ | SFB | 0.5 | ± | 0.1 | 4.0 | ± | 0.3 | 6.2 | ± | 0.3 | 10.2 | ± | 0.6 |
|  | CFB | 112.2 | ± | 7.1 | 0.0 | ± | 0.0 | 160.8 | ± | 8.1 | 15.1 | ± | 1.5 |

**^†^**LB = Lethbridge, Alberta; BL= Beaverlodge, Alberta; MB = Carman, Manitoba; SK = Saskatoon, Saskatchewan

^‡^ SFB = striped flea beetle; CFB = crucifer flea beetle

^§^ 2020: Lethbridge (AB); 2021, Beaverlodge (AB), MB, SK – different years due to different COVID-19 limitations

**Table S5.** Mean (± SE) flea beetles (FB) per sticky card per plant based on plot among 3 years, 3 plant densities, and 4 FB management treatments.

| **Year** | **Plant density** | **FB Management** | **LB^†^** | | | **BL** | | | **MB** | | | **SK** | | |
| --- | --- | --- | --- | --- | --- | --- | --- | --- | --- | --- | --- | --- | --- | --- |
| **2018** | Low | Control | 12.0 | ± | 1.7 | 1.1 | ± | 0.2 | 4.8 | ± | 0.6 | NA^‡^ | | |
|  |  | Foliar | 11.5 | ± | 3.3 | 0.9 | ± | 0.1 | 4.7 | ± | 0.6 |  |  |  |
|  |  | Seed | 8.5 | ± | 0.7 | 1.2 | ± | 0.1 | 5.7 | ± | 0.7 |  |  |  |
|  | Optimum | Control | 15.5 | ± | 4.0 | 1.1 | ± | 0.2 | 5.1 | ± | 0.9 | NA | | |
|  |  | Foliar | 16.0 | ± | 3.0 | 0.6 | ± | 0.1 | 3.3 | ± | 0.5 |  |  |  |
|  |  | Seed | 7.0 | ± | 0.6 | 0.6 | ± | 0.1 | 2.6 | ± | 0.2 |  |  |  |
|  | High | Control | 18.6 | ± | 2.5 | 0.7 | ± | 0.1 | 3.8 | ± | 0.6 | NA | | |
|  |  | Foliar | 16.2 | ± | 2.8 | 0.4 | ± | 0.1 | 3.2 | ± | 0.5 |  |  |  |
|  |  | Seed | 4.1 | ± | 1.0 | 0.6 | ± | 0.1 | 1.7 | ± | 0.2 |  |  |  |
| **2019** | Low | Control | 27.1 | ± | 3.7 | 4.3 | ± | 1.0 | 37.0 | ± | 10.1 | 6.3 | ± | 2.2 |
|  |  | Foliar | 28.5 | ± | 3.0 | 3.0 | ± | 0.5 | 21.1 | ± | 6.4 | 5.6 | ± | 2.4 |
|  |  | Seed | 46.8 | ± | 3.4 | 1.9 | ± | 0.1 | 20.4 | ± | 4.0 | 5.3 | ± | 1.8 |
|  |  | FB-Free | 26.9 | ± | 4.9 | 2.7 | ± | 0.6 | 20.1 | ± | 4.0 | NA | | |
|  | Optimum | Control | 28.9 | ± | 1.4 | 4.0 | ± | 2.4 | 24.4 | ± | 3.7 | 2.5 | ± | 0.8 |
|  |  | Foliar | 16.3 | ± | 1.0 | 2.4 | ± | 0.3 | 15.4 | ± | 3.6 | 2.0 | ± | 0.6 |
|  |  | Seed | 29.2 | ± | 3.8 | 1.1 | ± | 0.1 | 17.2 | ± | 4.8 | 3.4 | ± | 1.4 |
|  |  | FB-Free | 17.9 | ± | 2.4 | 1.8 | ± | 0.4 | 12.8 | ± | 2.3 | NA | | |
|  | High | Control | 17.1 | ± | 1.2 | 1.3 | ± | 0.1 | 14.1 | ± | 3.0 | 1.8 | ± | 0.5 |
|  |  | Foliar | 13.0 | ± | 3.7 | 1.6 | ± | 0.3 | 10.8 | ± | 3.1 | 1.5 | ± | 0.4 |
|  |  | Seed | 16.2 | ± | 2.2 | 0.9 | ± | 0.1 | 9.2 | ± | 1.9 | 1.3 | ± | 0.4 |
|  |  | FB-Free | 13.0 | ± | 2.1 | 1.1 | ± | 0.2 | 11.8 | ± | 3.5 | NA | | |
| **2020/2021**^⨎^ | Low | Control | 13.7 | ± | 3.4 | 0.6 | ± | 0.1 | 13.7 | ± | 1.4 | 1.5 | ± | 0.4 |
|  |  | Foliar | 11.7 | ± | 4.1 | 0.4 | ± | 0.0 | 11.3 | ± | 1.5 | 1.1 | ± | 0.2 |
|  |  | Seed | 11.3 | ± | 1.3 | 0.6 | ± | 0.1 | 25.3 | ± | 4.0 | 1.8 | ± | 0.2 |
|  |  | FB-Free | NA | | | 0.3 | ± | 0.1 | 9.0 | ± | 1.1 | 1.9 | ± | 0.5 |
|  | Optimum | Control | 22.1 | ± | 4.3 | 0.4 | ± | 0.1 | 11.9 | ± | 1.2 | 0.9 | ± | 0.1 |
|  |  | Foliar | 15.1 | ± | 5.3 | 0.2 | ± | 0.0 | 8.4 | ± | 0.9 | 0.9 | ± | 0.2 |
|  |  | Seed | 12.9 | ± | 0.7 | 0.6 | ± | 0.1 | 12.3 | ± | 1.9 | 1.4 | ± | 0.3 |
|  |  | FB-Free | NA | | | 0.5 | ± | 0.1 | 7.5 | ± | 1.0 | 0.5 | ± | 0.1 |
|  | High | Control | 7.2 | ± | 0.3 | 0.3 | ± | 0.0 | 5.8 | ± | 0.5 | 0.8 | ± | 0.2 |
|  |  | Foliar | 5.4 | ± | 2.2 | 0.3 | ± | 0.0 | 5.4 | ± | 0.5 | 0.9 | ± | 0.2 |
|  |  | Seed | 5.6 | ± | 0.6 | 0.5 | ± | 0.1 | 6.1 | ± | 0.8 | 0.8 | ± | 0.1 |
|  |  | FB-Free | NA | | | 0.2 | ± | 0.1 | 3.8 | ± | 0.4 | 0.5 | ± | 0.2 |

**^†^**LB = Lethbridge, Alberta; BL= Beaverlodge, Alberta; MB = Carman, Manitoba; SK = Saskatoon, Saskatchewan

‡ NAs indicate data missingness due to inability to access fields, collect data, or lack of personnel due to either COVID-19 limitations or other reasons preventing proper protocol

^⨎^ 2020: Lethbridge (AB); 2021, Beaverlodge (AB), MB, SK – different years due to different COVID-19 limitations

**Table S6.** Mean (± SE) defoliation (whole plant) (%) per plot among 3 years, 3 plant densities, and 4 flea beetle (FB) management treatments.

| **Year** | **Plant density** | **FB Management** | **LB^†^** | | | **BL** | | | **MB** | | | **SK** | | |
| --- | --- | --- | --- | --- | --- | --- | --- | --- | --- | --- | --- | --- | --- | --- |
| **2018** | Low | Control | 24.5 | ± | 0.8 | 18.8 | ± | 1.8 | 35.7 | ± | 1.6 | NA^‡^ | | |
|  |  | Foliar | 21.5 | ± | 2.4 | 15.6 | ± | 1.8 | 22.1 | ± | 0.9 |  |  |  |
|  |  | Seed | 12.3 | ± | 1.2 | 13.6 | ± | 1.9 | 16.3 | ± | 0.9 |  |  |  |
|  | Optimum | Control | 30.5 | ± | 5.5 | 20.3 | ± | 2.6 | 36.2 | ± | 1.4 | NA | | |
|  |  | Foliar | 28.2 | ± | 5.2 | 17.7 | ± | 2.1 | 21.8 | ± | 0.9 |  |  |  |
|  |  | Seed | 9.9 | ± | 1.1 | 11.0 | ± | 2.3 | 11.7 | ± | 1.1 |  |  |  |
|  | High | Control | 32.0 | ± | 3.7 | 19.2 | ± | 2.2 | 31.6 | ± | 1.5 | NA | | |
|  |  | Foliar | 27.8 | ± | 2.1 | 19.4 | ± | 2.9 | 21.0 | ± | 1.4 |  |  |  |
|  |  | Seed | 9.4 | ± | 1.7 | 10.3 | ± | 1.3 | 10.2 | ± | 1.3 |  |  |  |
| **2019** | Low | Control | 17.8 | ± | 3.0 | 26.6 | ± | 3.3 | 53.3 | ± | 2.9 | 20.6 | ± | 5.3 |
|  |  | Foliar | 13.9 | ± | 1.3 | 25.1 | ± | 3.3 | 25.9 | ± | 4.0 | 17.0 | ± | 3.9 |
|  |  | Seed | 12.9 | ± | 1.9 | 7.8 | ± | 2.2 | 21.5 | ± | 4.0 | 16.2 | ± | 3.9 |
|  |  | FB-Free | 9.9 | ± | 1.1 | 5.4 | ± | 0.8 | 29.0 | ± | 4.0 | NA | | |
|  | Optimum | Control | 22.6 | ± | 1.8 | 22.7 | ± | 3.2 | 52.9 | ± | 3.0 | 20.4 | ± | 5.3 |
|  |  | Foliar | 18.5 | ± | 1.0 | 18.9 | ± | 2.9 | 31.5 | ± | 3.9 | 14.1 | ± | 4.1 |
|  |  | Seed | 12.3 | ± | 1.4 | 11.1 | ± | 2.5 | 17.4 | ± | 2.6 | 11.8 | ± | 2.4 |
|  |  | FB-Free | 13.7 | ± | 1.6 | 7.6 | ± | 1.2 | 26.9 | ± | 4.6 | NA | | |
|  | High | Control | 22.7 | ± | 3.0 | 20.7 | ± | 4.1 | 46.8 | ± | 4.9 | 16.0 | ± | 4.1 |
|  |  | Foliar | 19.0 | ± | 1.2 | 13.9 | ± | 2.0 | 27.3 | ± | 2.9 | 12.5 | ± | 3.3 |
|  |  | Seed | 9.5 | ± | 1.8 | 10.3 | ± | 2.9 | 16.4 | ± | 3.5 | 9.9 | ± | 2.7 |
|  |  | FB-Free | 14.7 | ± | 1.4 | 9.9 | ± | 1.4 | 31.4 | ± | 4.2 | NA | | |
| **2020/2021**^⨎^ | Low | Control | 34.8 | ± | 1.6 | 8.5 | ± | 1.0 | 29.2 | ± | 7.6 | 22.1 | ± | 1.2 |
|  |  | Foliar | 32.0 | ± | 2.0 | 8.6 | ± | 1.0 | 23.4 | ± | 6.0 | 19.5 | ± | 1.4 |
|  |  | Seed | 18.5 | ± | 4.5 | 8.3 | ± | 0.5 | 11.9 | ± | 1.3 | 15.3 | ± | 1.2 |
|  |  | FB-Free | NA | | | 6.5 | ± | 1.2 | 19.6 | ± | 6.2 | 10.3 | ± | 2.5 |
|  | Optimum | Control | 40.1 | ± | 2.0 | 11.4 | ± | 2.5 | 28.7 | ± | 7.4 | 22.7 | ± | 1.3 |
|  |  | Foliar | 36.7 | ± | 1.6 | 8.2 | ± | 1.5 | 23.6 | ± | 5.5 | 16.0 | ± | 1.4 |
|  |  | Seed | 18.5 | ± | 1.4 | 7.6 | ± | 0.4 | 9.6 | ± | 1.6 | 12.2 | ± | 1.0 |
|  |  | FB-Free | NA | | | 10.7 | ± | 0.9 | 21.2 | ± | 6.3 | 6.0 | ± | 1.2 |
|  | High | Control | 43.9 | ± | 4.4 | 11.3 | ± | 1.8 | 25.7 | ± | 6.8 | 20.7 | ± | 2.6 |
|  |  | Foliar | 33.1 | ± | 4.3 | 8.8 | ± | 0.5 | 18.9 | ± | 4.5 | 17.3 | ± | 1.4 |
|  |  | Seed | 13.6 | ± | 1.5 | 6.7 | ± | 0.6 | 6.0 | ± | 0.9 | 10.1 | ± | 1.0 |
|  |  | FB-Free | NA | | | 8.1 | ± | 0.6 | 16.1 | ± | 5.1 | 7.8 | ± | 2.4 |

**^†^**LB = Lethbridge, Alberta; BL = Beaverlodge, Alberta; MB = Carman, Manitoba; SK = Saskatoon, Saskatchewan

‡ NAs indicate data missingness due to inability to access fields, collect data, or lack of personnel due to either COVID-19 limitations or other reasons preventing proper protocol

^⨎^ 2020: Lethbridge (AB); 2021, Beaverlodge (AB), MB, SK – different years due to different COVID-19 limitations

**Table S7.** Mean (± SE) yield (corrected at 8.5% moisture) (kg ha^-1^) per plot among 3 years, 3 plant densities, and 4 flea beetle (FB) management treatments

| **Year** | **Plant density** | **FB Management** | **LB^†^** | | | **BL** | | | **MB** | | | **SK** | | |
| --- | --- | --- | --- | --- | --- | --- | --- | --- | --- | --- | --- | --- | --- | --- |
| **2018** | Low | Control | 661.4 | ± | 125.7 | 2985.1 | ± | 165.6 | 1698.4 | ± | 154.1 | NA^‡^ | | |
|  |  | Foliar | 811.6 | ± | 71.2 | 2544.1 | ± | 450.7 | 2158.3 | ± | 210.1 |  |  |  |
|  |  | Seed | 920.9 | ± | 26.7 | 3295.9 | ± | 167.7 | 2250.5 | ± | 220.3 |  |  |  |
|  | Optimum | Control | 856.9 | ± | 38.1 | 3326.3 | ± | 303.6 | 2013.3 | ± | 186.7 | NA | | |
|  |  | Foliar | 922.4 | ± | 54.2 | 3480.7 | ± | 122.0 | 2134.0 | ± | 210.9 |  |  |  |
|  |  | Seed | 1004.4 | ± | 13.1 | 3630.8 | ± | 163.1 | 2405.8 | ± | 242.8 |  |  |  |
|  | High | Control | 955.6 | ± | 32.4 | 3463.7 | ± | 323.2 | 2188.2 | ± | 255.6 | NA | | |
|  |  | Foliar | 996.9 | ± | 21.0 | 3747.0 | ± | 119.4 | 2507.8 | ± | 247.8 |  |  |  |
|  |  | Seed | 976.5 | ± | 29.6 | 3792.8 | ± | 318.0 | 2090.2 | ± | 163.1 |  |  |  |
| **2019** | Low | Control | 652.5 | ± | 76.9 | 2596.1 | ± | 366.7 | 215.8 | ± | 40.8 | 1978.2 | ± | 385.3 |
|  |  | Foliar | 831.2 | ± | 176.3 | 3354.8 | ± | 293.5 | 870.0 | ± | 66.7 | 3424.6 | ± | 141.4 |
|  |  | Seed | 725.0 | ± | 84.1 | 3123.2 | ± | 150.3 | 819.1 | ± | 98.1 | 2111.9 | ± | 345.1 |
|  |  | FB-Free | 699.7 | ± | 88.1 | 3629.9 | ± | 300.9 | 774.3 | ± | 49.3 | NA | | |
|  | Optimum | Control | 685.3 | ± | 94.8 | 3287.7 | ± | 276.8 | 378.7 | ± | 81.8 | 2891.5 | ± | 232.3 |
|  |  | Foliar | 780.6 | ± | 75.0 | 3388.1 | ± | 396.7 | 895.1 | ± | 68.2 | 3913.6 | ± | 215.4 |
|  |  | Seed | 736.8 | ± | 109.6 | 3304.7 | ± | 384.4 | 882.5 | ± | 61.3 | 3161.9 | ± | 160.3 |
|  |  | FB-Free | 685.3 | ± | 58.9 | 3857.2 | ± | 259.6 | 804.1 | ± | 65.1 | NA | | |
|  | High | Control | 817.7 | ± | 116.2 | 3801.9 | ± | 157.0 | 608.1 | ± | 61.3 | 2668.2 | ± | 171.8 |
|  |  | Foliar | 595.7 | ± | 48.8 | 3625.5 | ± | 355.8 | 1010.5 | ± | 63.2 | 3321.5 | ± | 195.7 |
|  |  | Seed | 708.0 | ± | 34.1 | 3368.0 | ± | 137.7 | 840.8 | ± | 70.2 | 2986.0 | ± | 136.1 |
|  |  | FB-Free | 802.4 | ± | 87.9 | 3849.0 | ± | 316.2 | 973.9 | ± | 52.5 | NA | | |
| **2020/2021**^⨎^ | Low | Control | 106.9 | ± | 29.0 | 783.9 | ± | 223.3 | 106.8 | ± | 32.0 | 567.6 | ± | 68.5 |
|  |  | Foliar | 99.7 | ± | 11.1 | 628.8 | ± | 173.7 | 285.4 | ± | 112.6 | 748.7 | ± | 58.6 |
|  |  | Seed | 230.1 | ± | 37.0 | 1049.5 | ± | 162.9 | 192.9 | ± | 74.4 | 753.6 | ± | 64.4 |
|  |  | FB-Free | NA | | | 441.7 | ± | 124.1 | 162.5 | ± | 87.4 | 698.0 | ± | 78.3 |
|  | Optimum | Control | 163.5 | ± | 7.9 | 855.6 | ± | 193.7 | 140.0 | ± | 57.5 | 722.2 | ± | 93.4 |
|  |  | Foliar | 217.9 | ± | 45.8 | 1139.6 | ± | 332.1 | 378.8 | ± | 94.5 | 1212.5 | ± | 105.1 |
|  |  | Seed | 392.6 | ± | 31.8 | 973.5 | ± | 210.5 | 290.5 | ± | 101.6 | 884.0 | ± | 65.6 |
|  |  | FB-Free | NA | | | 969.8 | ± | 249.6 | 231.2 | ± | 125.7 | 700.4 | ± | 52.7 |
|  | High | Control | 231.1 | ± | 37.1 | 738.3 | ± | 122.9 | 211.6 | ± | 136.6 | 741.5 | ± | 60.1 |
|  |  | Foliar | 341.2 | ± | 10.5 | 630.7 | ± | 151.8 | 487.4 | ± | 115.8 | 1268.0 | ± | 76.7 |
|  |  | Seed | 457.5 | ± | 35.9 | 781.2 | ± | 176.9 | 357.6 | ± | 159.7 | 900.9 | ± | 53.4 |
|  |  | FB-Free | NA | | | 870.7 | ± | 173.6 | 439.1 | ± | 194.3 | 954.0 | ± | 109.2 |

**^†^**LB = Lethbridge, Alberta; BL= Beaverlodge, Alberta; MB = Carman, Manitoba; SK = Saskatoon, Saskatchewan

‡ NAs indicate data missingness due to inability to access fields, collect data, or lack of personnel due to either COVID-19 limitations or other reasons preventing proper protocol

^⨎^ 2020: Lethbridge (AB); 2021, Beaverlodge (AB), MB, SK – different years due to different COVID-19 limitations

**Table S8**. Mean (± SE) plants per square metre, yield (corrected at 8.5% moisture) (kg ha^-1^), and defoliation (%) per trial in each region among 3 years, 3 plant densities, and 4 flea beetle (FB) management treatments

| **Year** | | **Region** | **Trial** | | **Plant density** | **FB Management** | **Plants/m^2^** | | | **Yield (kg ha^-1^)** | | | **Mean Defoliation (%)** | | |
| --- | --- | --- | --- | --- | --- | --- | --- | --- | --- | --- | --- | --- | --- | --- | --- |
| **2018** | Lethbridge, AB | | | 1 | Low | Control ^†^ | 28.84 | ± | 4.58 | 672.41 | ± | 127.78 | 24.54 | ± | 0.82 |
|  |  | | |  |  | Foliar | 32.59 | ± | 6.23 | 825.11 | ± | 72.41 | 21.52 | ± | 2.39 |
|  |  | | |  |  | Seed | 37.37 | ± | 1.65 | 936.21 | ± | 27.19 | 12.26 | ± | 1.15 |
|  |  | | |  | Optimum | Control ^†^ | 55.99 | ± | 2.59 | 871.23 | ± | 38.76 | 30.47 | ± | 5.45 |
|  |  | | |  |  | Foliar ^†^ | 59.74 | ± | 2.94 | 937.82 | ± | 55.06 | 28.16 | ± | 5.21 |
|  |  | | |  |  | Seed | 62.33 | ± | 2.97 | 1021.12 | ± | 13.28 | 9.91 | ± | 1.10 |
|  |  | | |  | High | Control ^†^ | 100.73 | ± | 10.05 | 971.55 | ± | 32.90 | 32.05 | ± | 3.73 |
|  |  | | |  |  | Foliar ^†^ | 119.87 | ± | 12.11 | 1013.47 | ± | 21.31 | 27.84 | ± | 2.11 |
|  |  | | |  |  | Seed | 108.49 | ± | 14.69 | 992.78 | ± | 30.06 | 9.39 | ± | 1.68 |
|  | Beaverlodge, AB | | | 1 | Low | Control | 31.11 | ± | 1.97 | 3217.85 | ± | 170.07 | 18.79 | ± | 1.84 |
|  |  | | |  |  | Foliar | 26.85 | ± | 0.81 | 2736.20 | ± | 482.91 | 15.61 | ± | 1.79 |
|  |  | | |  |  | Seed | 30.28 | ± | 4.28 | 3530.35 | ± | 172.41 | 13.62 | ± | 1.86 |
|  |  | | |  | Optimum | Control | 46.02 | ± | 7.93 | 3563.58 | ± | 315.16 | 20.33 | ± | 2.60 |
|  |  | | |  |  | Foliar | 55.46 | ± | 5.69 | 3708.82 | ± | 120.42 | 17.72 | ± | 2.06 |
|  |  | | |  |  | Seed | 61.11 | ± | 4.35 | 3847.54 | ± | 174.57 | 11.01 | ± | 2.26 |
|  |  | | |  | High | Control | 87.30 | ± | 6.41 | 3676.30 | ± | 341.66 | 19.19 | ± | 2.24 |
|  |  | | |  |  | Foliar | 100.28 | ± | 5.72 | 3960.26 | ± | 114.33 | 19.41 | ± | 2.86 |
|  |  | | |  |  | Seed | 91.30 | ± | 6.98 | 3989.16 | ± | 327.36 | 10.27 | ± | 1.27 |
|  | Carman, MB | | | 1 | Low | Control ^†^ | 48.13 | ± | 0.69 | 1760.11 | ± | 295.74 | 35.28 | ± | 2.49 |
|  |  | | |  |  | Foliar | 58.04 | ± | 6.11 | 2556.08 | ± | 133.97 | 22.27 | ± | 0.50 |
|  |  | | |  |  | Seed | 53.52 | ± | 4.13 | 2614.92 | ± | 119.53 | 16.57 | ± | 1.53 |
|  |  | | |  | Optimum | Control ^†^ | 91.69 | ± | 5.71 | 2342.25 | ± | 151.18 | 35.32 | ± | 2.57 |
|  |  | | |  |  | Foliar | 101.94 | ± | 5.79 | 2387.42 | ± | 271.57 | 21.10 | ± | 1.67 |
|  |  | | |  |  | Seed | 110.83 | ± | 3.77 | 2837.25 | ± | 124.24 | 11.63 | ± | 1.54 |
|  |  | | |  | High | Control ^†^ | 195.24 | ± | 20.60 | 2599.79 | ± | 196.25 | 30.68 | ± | 2.10 |
|  |  | | |  |  | Foliar | 202.42 | ± | 9.04 | 2816.13 | ± | 278.36 | 19.25 | ± | 1.74 |
|  |  | | |  |  | Seed | 206.79 | ± | 15.06 | 2071.88 | ± | 177.82 | 9.03 | ± | 2.33 |
|  |  | | | 2 | Low | Control ^†^ | 54.39 | ± | 2.49 | 1557.75 | ± | 168.33 | 35.33 | ± | 2.74 |
|  |  | | |  |  | Foliar | 54.83 | ± | 3.57 | 1623.50 | ± | 173.64 | 21.90 | ± | 1.80 |
|  |  | | |  |  | Seed | 50.67 | ± | 6.53 | 1743.17 | ± | 267.07 | 16.08 | ± | 1.07 |
|  |  | | |  | Optimum | Control ^†^ | 83.28 | ± | 4.48 | 1556.54 | ± | 163.64 | 37.18 | ± | 1.25 |
|  |  | | |  |  | Foliar | 110.60 | ± | 8.77 | 1745.04 | ± | 228.00 | 22.45 | ± | 0.76 |
|  |  | | |  |  | Seed | 94.57 | ± | 3.86 | 1821.67 | ± | 265.52 | 11.67 | ± | 1.79 |
|  |  | | |  | High | Control ^†^ | 176.40 | ± | 14.69 | 1637.58 | ± | 304.80 | 32.58 | ± | 2.28 |
|  |  | | |  |  | Foliar | 175.97 | ± | 7.13 | 2040.17 | ± | 301.31 | 22.81 | ± | 1.94 |
|  |  | | |  |  | Seed | 176.81 | ± | 4.75 | 1975.75 | ± | 288.42 | 11.41 | ± | 1.07 |
| **2019** | Lethbridge, AB | | | 1 | Low | Control | 39.46 | ± | 5.43 | 663.36 | ± | 78.15 | 17.83 | ± | 3.00 |
|  |  | | |  |  | Foliar | 36.00 | ± | 3.45 | 737.07 | ± | 85.55 | 13.88 | ± | 1.35 |
|  |  | | |  |  | Seed | 27.62 | ± | 1.97 | 711.31 | ± | 89.60 | 12.93 | ± | 1.86 |
|  |  | | |  |  | FB-Free | 36.21 | ± | 5.18 | 845.04 | ± | 179.27 | 9.92 | ± | 1.11 |
|  |  | | |  | Optimum | Control | 51.00 | ± | 5.08 | 696.77 | ± | 96.37 | 22.63 | ± | 1.78 |
|  |  | | |  |  | Foliar | 56.28 | ± | 2.30 | 749.03 | ± | 111.47 | 18.49 | ± | 0.95 |
|  |  | | |  |  | Seed | 46.55 | ± | 3.79 | 696.77 | ± | 59.85 | 12.32 | ± | 1.43 |
|  |  | | |  |  | FB-Free | 53.79 | ± | 2.41 | 793.64 | ± | 76.24 | 13.74 | ± | 1.62 |
|  |  | | |  | High | Control | 88.34 | ± | 4.23 | 831.36 | ± | 118.10 | 22.67 | ± | 3.01 |
|  |  | | |  |  | Foliar | 105.83 | ± | 12.41 | 719.83 | ± | 34.69 | 19.04 | ± | 1.22 |
|  |  | | |  |  | Seed | 72.72 | ± | 3.61 | 815.73 | ± | 89.40 | 9.49 | ± | 1.80 |
|  |  | | |  |  | FB-free | 98.90 | ± | 4.42 | 605.60 | ± | 49.65 | 14.65 | ± | 1.39 |
|  | Beaverlodge, AB | | | 1 | Low | Control^†^ | 23.07 | ± | 2.83 | 2892.34 | ± | 394.39 | 26.62 | ± | 3.34 |
|  |  | | |  |  | Foliar^†^ | 35.34 | ± | 5.51 | 3492.05 | ± | 172.45 | 25.08 | ± | 3.35 |
|  |  | | |  |  | Seed | 33.07 | ± | 5.07 | 4036.13 | ± | 340.65 | 7.85 | ± | 2.24 |
|  |  | | |  |  | FB-Free | 29.79 | ± | 3.90 | 3711.78 | ± | 328.18 | 5.38 | ± | 0.83 |
|  |  | | |  | Optimum | Control | 61.75 | ± | 5.12 | 3654.41 | ± | 308.73 | 22.66 | ± | 3.16 |
|  |  | | |  |  | Foliar | 51.64 | ± | 6.18 | 3663.66 | ± | 430.98 | 18.86 | ± | 2.93 |
|  |  | | |  |  | Seed | 66.67 | ± | 5.17 | 4261.56 | ± | 298.40 | 11.13 | ± | 2.46 |
|  |  | | |  |  | FB-Free | 63.28 | ± | 6.76 | 3719.73 | ± | 445.96 | 7.55 | ± | 1.18 |
|  |  | | |  | High | Control | 107.62 | ± | 2.91 | 4164.74 | ± | 179.32 | 20.73 | ± | 4.14 |
|  |  | | |  |  | Foliar | 102.28 | ± | 18.52 | 3677.02 | ± | 154.79 | 13.90 | ± | 2.02 |
|  |  | | |  |  | Seed | 113.81 | ± | 14.82 | 4212.43 | ± | 344.55 | 10.27 | ± | 2.91 |
|  |  | | |  |  | FB-free | 113.49 | ± | 16.63 | 3964.60 | ± | 401.41 | 9.88 | ± | 1.38 |
|  | Saskatoon, SK | | | 1 | Low | Control^†^ | 14.47 | ± | 3.70 | 1365.84 | ± | 513.09 | 33.29 | ± | 4.74 |
|  |  | | |  |  | Foliar^†^ | 14.92 | ± | 5.31 | 1556.43 | ± | 450.79 | 26.45 | ± | 3.08 |
|  |  | | |  |  | Seed^†^ | 22.25 | ± | 3.35 | 3054.93 | ± | 247.12 | 26.30 | ± | 0.92 |
|  |  | | |  | Optimum | Control^†^ | 49.75 | ± | 7.72 | 2974.59 | ± | 315.94 | 34.07 | ± | 2.42 |
|  |  | | |  |  | Foliar | 63.75 | ± | 9.42 | 3099.78 | ± | 259.75 | 23.67 | ± | 3.93 |
|  |  | | |  |  | Seed | 25.83 | ± | 4.79 | 2283.26 | ± | 670.81 | 17.62 | ± | 2.10 |
|  |  | | |  | High | Control^†^ | 97.17 | ± | 10.25 | 2866.22 | ± | 164.50 | 24.72 | ± | 5.21 |
|  |  | | |  |  | Foliar | 99.42 | ± | 4.77 | 3340.81 | ± | 64.80 | 19.03 | ± | 4.55 |
|  |  | | |  |  | Seed | 84.00 | ± | 8.25 | 3456.65 | ± | 334.32 | 15.95 | ± | 3.18 |
|  |  | | | 2 | Low | Control | 39.00 | ± | 7.17 | 3198.80 | ± | 334.91 | 7.92 | ± | 0.73 |
|  |  | | |  |  | Foliar | 52.89 | ± | 3.98 | 3146.49 | ± | 338.53 | 7.51 | ± | 1.33 |
|  |  | | |  |  | Seed | 54.18 | ± | 11.33 | 3404.33 | ± | 168.93 | 6.02 | ± | 0.54 |
|  |  | | |  | Optimum | Control | 75.78 | ± | 9.41 | 3381.91 | ± | 155.98 | 6.77 | ± | 0.49 |
|  |  | | |  |  | Foliar | 68.11 | ± | 8.23 | 3576.23 | ± | 153.94 | 4.57 | ± | 0.66 |
|  |  | | |  |  | Seed | 55.44 | ± | 5.04 | 3572.50 | ± | 216.44 | 6.07 | ± | 0.83 |
|  |  | | |  | High | Control | 108.43 | ± | 19.14 | 2810.16 | ± | 270.09 | 7.34 | ± | 0.64 |
|  |  | | |  |  | Foliar | 98.89 | ± | 11.65 | 2959.64 | ± | 159.95 | 6.02 | ± | 0.94 |
|  |  | | |  |  | Seed | 111.00 | ± | 7.83 | 3471.60 | ± | 194.36 | 3.94 | ± | 0.95 |
|  | Carman, MB | | | 1 | Low | Control^†^ | 23.24 | ± | 2.92 | 131.14 | ± | 33.95 | 56.78 | ± | 3.79 |
|  |  | | |  |  | Foliar^†^ | 32.96 | ± | 3.09 | 715.08 | ± | 97.88 | 30.29 | ± | 4.50 |
|  |  | | |  |  | Seed^†^ | 36.75 | ± | 4.05 | 494.79 | ± | 177.47 | 27.22 | ± | 6.78 |
|  |  | | |  |  | FB-Free^†^ | 34.61 | ± | 4.08 | 686.58 | ± | 50.96 | 36.61 | ± | 3.16 |
|  |  | | |  | Optimum | Control^†^ | 49.39 | ± | 4.84 | 175.44 | ± | 53.08 | 59.58 | ± | 0.82 |
|  |  | | |  |  | Foliar^†^ | 63.93 | ± | 5.22 | 772.94 | ± | 77.24 | 37.35 | ± | 5.19 |
|  |  | | |  |  | Seed | 56.49 | ± | 6.10 | 758.14 | ± | 82.49 | 23.47 | ± | 2.20 |
|  |  | | |  |  | FB-Free^†^ | 70.94 | ± | 6.62 | 898.03 | ± | 81.42 | 34.77 | ± | 6.29 |
|  |  | | |  | High | Control^†^ | 110.62 | ± | 10.77 | 562.31 | ± | 113.16 | 58.29 | ± | 4.31 |
|  |  | | |  |  | Foliar^†^ | 127.26 | ± | 18.18 | 837.25 | ± | 94.34 | 25.02 | ± | 5.70 |
|  |  | | |  |  | Seed | 108.05 | ± | 8.72 | 884.39 | ± | 65.44 | 24.44 | ± | 3.75 |
|  |  | | |  |  | FB-free^†^ | 110.37 | ± | 17.78 | 956.89 | ± | 53.16 | 40.87 | ± | 4.04 |
|  |  | | | 2 | Low | Control^†^ | 32.33 | ± | 3.04 | 286.75 | ± | 45.68 | 49.86 | ± | 4.00 |
|  |  | | |  |  | Foliar | 42.17 | ± | 2.16 | 871.17 | ± | 168.59 | 21.53 | ± | 6.39 |
|  |  | | |  |  | Seed | 33.93 | ± | 4.00 | 805.00 | ± | 50.26 | 15.83 | ± | 2.46 |
|  |  | | |  |  | FB-Free | 35.58 | ± | 3.30 | 998.22 | ± | 26.40 | 21.34 | ± | 5.15 |
|  |  | | |  | Optimum | Control^†^ | 62.47 | ± | 3.01 | 557.89 | ± | 45.70 | 46.32 | ± | 3.55 |
|  |  | | |  |  | Foliar^†^ | 70.88 | ± | 3.92 | 936.03 | ± | 77.89 | 25.74 | ± | 4.83 |
|  |  | | |  |  | Seed | 84.19 | ± | 20.63 | 798.97 | ± | 107.06 | 11.35 | ± | 1.44 |
|  |  | | |  |  | FB-Free | 60.08 | ± | 8.64 | 835.36 | ± | 114.18 | 19.04 | ± | 4.04 |
|  |  | | |  | High | Control^†^ | 116.11 | ± | 5.12 | 615.25 | ± | 56.14 | 35.32 | ± | 2.22 |
|  |  | | |  |  | Foliar^†^ | 124.75 | ± | 10.42 | 790.92 | ± | 110.91 | 29.51 | ± | 1.54 |
|  |  | | |  |  | Seed | 98.83 | ± | 9.12 | 1001.58 | ± | 73.94 | 8.30 | ± | 0.61 |
|  |  | | |  |  | FB-free | 126.51 | ± | 5.87 | 999.89 | ± | 119.75 | 21.86 | ± | 2.76 |
| **2020** | Lethbridge, AB | | | 1 | Low | Control^†^ | NA | | | 108.73 | ± | 29.52 | 34.79 | ± | 1.56 |
|  |  | | |  |  | Foliar^†^ |  |  |  | 101.31 | ± | 11.30 | 32.05 | ± | 2.04 |
|  |  | | |  |  | Seed |  |  |  | 233.93 | ± | 37.60 | 18.49 | ± | 4.47 |
|  |  | | |  | Optimum | Control^†^ | NA | | | 166.24 | ± | 7.99 | 40.08 | ± | 1.95 |
|  |  | | |  |  | Foliar^†^ |  |  |  | 221.49 | ± | 46.59 | 36.70 | ± | 1.56 |
|  |  | | |  |  | Seed |  |  |  | 399.11 | ± | 32.34 | 18.52 | ± | 1.38 |
|  |  | | |  | High | Control^†^ | NA | | | 234.97 | ± | 37.68 | 43.93 | ± | 4.44 |
|  |  | | |  |  | Foliar^†^ |  |  |  | 346.85 | ± | 10.67 | 33.13 | ± | 4.35 |
|  |  | | |  |  | Seed |  |  |  | 465.10 | ± | 36.49 | 13.65 | ± | 1.55 |
| **2021** | Beaverlodge, AB | | | 1 | Low | Control | 26.36 | ± | 1.47 | 811.71 | ± | 232.74 | 8.52 | ± | 0.96 |
|  |  | | |  |  | Foliar | 26.30 | ± | 5.95 | 1071.89 | ± | 166.36 | 8.57 | ± | 0.96 |
|  |  | | |  |  | Seed | 15.40 | ± | 1.49 | 454.99 | ± | 129.88 | 8.25 | ± | 0.46 |
|  |  | | |  |  | FB-Free | 19.88 | ± | 2.36 | 642.70 | ± | 175.67 | 6.49 | ± | 1.20 |
|  |  | | |  | Optimum | Control | 44.04 | ± | 7.32 | 882.01 | ± | 200.85 | 11.36 | ± | 2.54 |
|  |  | | |  |  | Foliar | 53.70 | ± | 6.99 | 983.82 | ± | 214.52 | 8.18 | ± | 1.54 |
|  |  | | |  |  | Seed | 30.43 | ± | 2.84 | 1007.37 | ± | 257.25 | 7.58 | ± | 0.36 |
|  |  | | |  |  | FB-Free | 48.21 | ± | 8.85 | 1159.03 | ± | 342.50 | 10.67 | ± | 0.94 |
|  |  | | |  | High | Control | 111.59 | ± | 10.42 | 756.58 | ± | 123.71 | 11.29 | ± | 1.82 |
|  |  | | |  |  | Foliar | 105.31 | ± | 10.63 | 799.78 | ± | 181.09 | 8.79 | ± | 0.53 |
|  |  | | |  |  | Seed | 57.65 | ± | 5.75 | 890.90 | ± | 176.82 | 6.74 | ± | 0.63 |
|  |  | | |  |  | FB-free | 87.04 | ± | 15.06 | 640.75 | ± | 152.95 | 8.09 | ± | 0.65 |
|  | Saskatoon, SK | | | 1 | Low | Control | 34.07 | ± | 2.14 | 587.50 | ± | 70.87 | 22.15 | ± | 1.23 |
|  |  | | |  |  | Foliar | 48.20 | ± | 2.85 | 780.00 | ± | 66.71 | 19.49 | ± | 1.38 |
|  |  | | |  |  | Seed | 33.47 | ± | 4.50 | 722.50 | ± | 81.07 | 15.32 | ± | 1.22 |
|  |  | | |  |  | FB-Free | 38.09 | ± | 5.14 | 775.00 | ± | 60.62 | 10.30 | ± | 2.48 |
|  |  | | |  | Optimum | Control | 72.27 | ± | 3.73 | 747.50 | ± | 96.64 | 22.73 | ± | 1.29 |
|  |  | | |  |  | Foliar | 69.67 | ± | 3.00 | 915.00 | ± | 67.88 | 16.02 | ± | 1.40 |
|  |  | | |  |  | Seed | 45.33 | ± | 3.46 | 725.00 | ± | 54.54 | 12.24 | ± | 1.03 |
|  |  | | |  |  | FB-Free | 77.67 | ± | 2.53 | 1255.00 | ± | 108.82 | 6.05 | ± | 1.21 |
|  |  | | |  | High | Control | 132.00 | ± | 5.25 | 767.50 | ± | 62.23 | 20.71 | ± | 2.57 |
|  |  | | |  |  | Foliar | 119.53 | ± | 11.39 | 932.50 | ± | 55.28 | 17.32 | ± | 1.40 |
|  |  | | |  |  | Seed | 93.00 | ± | 3.81 | 987.50 | ± | 113.02 | 10.14 | ± | 1.01 |
|  |  | | |  |  | FB-free | 143.03 | ± | 14.62 | 1312.50 | ± | 79.41 | 7.80 | ± | 2.44 |
|  | Carman, MB | | | 1 | Low | Control^†^ | 35.35 | ± | 1.86 | 119.80 | ± | 39.21 | 48.79 | ± | 3.66 |
|  |  | | |  |  | Foliar^†^ | 37.22 | ± | 1.31 | 282.13 | ± | 123.85 | 38.31 | ± | 4.20 |
|  |  | | |  |  | Seed | 32.43 | ± | 2.57 | 286.93 | ± | 148.39 | 14.97 | ± | 1.18 |
|  |  | | |  |  | FB-Free^†^ | 38.24 | ± | 4.16 | 315.47 | ± | 221.72 | 34.04 | ± | 6.29 |
|  |  | | |  | Optimum | Control^†^ | 56.58 | ± | 4.68 | 191.60 | ± | 99.68 | 47.60 | ± | 4.07 |
|  |  | | |  |  | Foliar^†^ | 69.18 | ± | 8.27 | 477.30 | ± | 123.87 | 36.99 | ± | 4.57 |
|  |  | | |  |  | Seed | 65.68 | ± | 13.00 | 363.87 | ± | 232.15 | 12.25 | ± | 2.55 |
|  |  | | |  |  | FB-Free^†^ | 64.17 | ± | 6.55 | 457.53 | ± | 155.71 | 37.06 | ± | 4.22 |
|  |  | | |  | High | Control^†^ | 119.82 | ± | 19.03 | 316.87 | ± | 257.92 | 43.51 | ± | 1.75 |
|  |  | | |  |  | Foliar^†^ | 112.23 | ± | 2.11 | 510.63 | ± | 277.56 | 29.78 | ± | 4.14 |
|  |  | | |  |  | Seed | 162.40 | ± | 13.94 | 688.50 | ± | 323.48 | 6.51 | ± | 1.67 |
|  |  | | |  |  | FB-Free^†^ | 107.57 | ± | 10.56 | 598.40 | ± | 180.81 | 28.78 | ± | 3.48 |
|  |  | | | 2 | Low | Control | 97.30 | ± | 7.07 | 86.97 | ± | 52.61 | 9.59 | ± | 1.70 |
|  |  | | |  |  | Foliar | 86.45 | ± | 3.98 | 91.43 | ± | 52.61 | 8.55 | ± | 1.25 |
|  |  | | |  |  | Seed | 32.20 | ± | 2.88 | 27.50 | ± | 6.50 | 8.92 | ± | 0.78 |
|  |  | | |  |  | FB-Free | 76.88 | ± | 2.30 | 236.70 | ± | 70.92 | 5.10 | ± | 0.68 |
|  |  | | |  | Optimum | Control | 84.35 | ± | 4.87 | 79.67 | ± | 49.64 | 9.84 | ± | 0.64 |
|  |  | | |  |  | Foliar | 87.73 | ± | 2.06 | 85.13 | ± | 64.54 | 10.19 | ± | 1.59 |
|  |  | | |  |  | Seed | 101.97 | ± | 1.88 | 83.83 | ± | 46.36 | 6.92 | ± | 0.85 |
|  |  | | |  |  | FB-Free | 96.83 | ± | 4.78 | 276.17 | ± | 96.66 | 5.38 | ± | 0.95 |
|  |  | | |  | High | Control | 167.07 | ± | 4.09 | 92.43 | ± | 79.74 | 7.80 | ± | 0.47 |
|  |  | | |  |  | Foliar | 172.32 | ± | 3.45 | 181.50 | ± | 126.82 | 8.08 | ± | 0.91 |
|  |  | | |  |  | Seed | 186.55 | ± | 11.08 | 162.63 | ± | 120.54 | 5.55 | ± | 0.68 |
|  |  | | |  |  | FB-free | 170.33 | ± | 7.88 | 346.10 | ± | 124.53 | 3.35 | ± | 0.40 |

^†^ trials that exceeded the economic threshold of 25% defoliation

**Fig. S1** Mean (± SE) scaled log transformed abundances of (A) total SFB/sticky card and (B) total CFB/sticky card based on PD and FB management treatment for all regions combined. Capital letters represent differences among PD treatments within each FB management treatment/ region. Lower-case letters represent differences among FB management treatments/ region within each PD treatment (EMM, ⍺ = 0.05). FB = flea beetle; PD = plant density; SFB = striped flea beetle, CFB = crucifer flea beetle.

**Fig. S2** Average number of plants per linear metre in each FB management treatment, and plant density for a) Lethbridge, AB; b) Beaverlodge, AB; c) Saskatoon, SK; and d) Carman, MB. Data taken from 50% plant emergence to growth stage 2.4. Letters indicate significant differences among FB management treatments within each PD per region (EMM, ⍺ = 0.05). Non-significant differences among PD are denoted with *n.s.* within each PD treatment (EMM, ⍺ = 0.05). FB *=* flea beetle; PD = plant density*;* SFB = striped flea beetle, CFB = crucifer flea beetle.

***n.s.***
